# Neurophilic Biomimetic Lipoprotein‐Mediated Targeted Nerve Growth Factor Delivery for Traumatic Brain Injury Therapy

**DOI:** 10.1002/advs.202509405

**Published:** 2025-08-18

**Authors:** Jialin Huang, Wenye Wang, Yidong Peng, Weiji Weng, Hanyu Wei, Qiyuan Feng, Antian Wang, Minjie Hu, Zhuoran Li, Shenyu Sun, Zhenghui He, Daiwen Zhang, Wenlan Qi, Yuhan Han, Zixuan Ma, Jiyuan Hui, Ru Gong, Yingwei Gao, Yong Lin, Jiyao Jiang, Xiaoling Gao, Junfeng Feng

**Affiliations:** ^1^ Department of Neurosurgery Renji Hospital Shanghai Jiao Tong University School of Medicine Shanghai 200127 China; ^2^ Shanghai Institute of Head Trauma Shanghai 200127 China; ^3^ Department of Pharmacology and Chemical Biology Shanghai Universities Collaborative Innovation Center for Translational Medicine Shanghai Jiao Tong University School of Medicine Shanghai 200025 China; ^4^ Department of Radiation Oncology The First Hospital of Lanzhou University Lanzhou University Lanzhou 730000 China

**Keywords:** neural regeneration and repair, neurophilic biomimetic lipoprotein, NGF delivery, rHDL, traumatic brain injury

## Abstract

Traumatic brain injury (TBI) induces neuronal death, inflammation, and neurological dysfunction. Although nerve growth factor (NGF) possesses neuroprotective potential, its clinical use is hindered by poor blood‐brain barrier (BBB) permeability and insufficient neural targeting. Here, a neurophilic biomimetic lipoprotein for brain‐targeted NGF delivery is developed: 1) a matrix‐like core (Nc) that preserves NGF bioactivity; 2) an ApoE3‐reconstituted high‐density lipoprotein shell (Nc‐rHDL) to enhance BBB penetration; 3) an αRDP peptide‐modified version (Nc‐rHDL@P) to improve neural targeting. In vitro BBB models and controlled cortical impact (CCI) mice demonstrate that Nc‐rHDL@P efficiently crossed the BBB and selectively accumulated around injured regions. The engineered Nc‐rHDL@P significantly enhances the survival of injured neurons, promotes neurite outgrowth in PC12 cells, and facilitates the neuronal differentiation of human neural stem cells (hNSCs) and Schwann cells in vitro. In vivo studies confirm that Nc‐rHDL@P effectively alleviated inflammation and glial scar formation while significantly increasing neuronal survival—ultimately facilitating the recovery of motor function, spatial learning, and memory in CCI model mice. Collectively, this neurophilic biomimetic lipoprotein platform demonstrates broad potential for brain‐targeted delivery of neurotrophins beyond NGF, offering a promising translational strategy for TBI and related neurological disorders.

## Graphical abstract

Design of neurophilic biomimetic lipoprotein for intracerebral delivery of nerve growth factor (NGF) to traumatic brain injury (TBI). Nc‐rHDL@P can cross the BBB through ApoE3‐receptors interaction and target neurons via αRDP‐nAChR interaction. Nc‐rHDL@P delivers NGF to the lesion sites, rescues neurons, curbs inflammation, boosts sensorimotor and memory in mice. The graphic abstract is created in BioRender. Weng, W. (2025) (https://BioRender.com/25xnrv1).

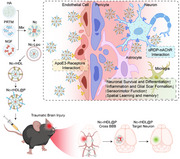

## 1 Introduction

Traumatic brain injury (TBI) is one of the leading causes of injury‐related death and disability all over the world.^[^ ^1^, ^2^ ^]^ External forces can inflict damage on the brain's structure and function, thereby inducing a range of physical, cognitive, and behavioral symptoms.^[^ ^2^, ^3^, ^4^ ^]^ Currently, there are no effective pharmaceuticals available in clinics to treat TBI.^[^ ^5^, ^6^, ^7^ ^]^ It is essential to develop neuroprotective therapeutics to mitigate detrimental outcomes following TBI to improve cognitive and behavioral functioning.

In the early 1950s, nerve growth factor (NGF) was identified as the first member of the neurotrophin family, known for its important regulatory functions on the survival, growth, and differentiation of nerve cells.^[^ ^8^ ^]^ However, following TBI, the endogenous expression and secretion of NGF decrease significantly in the lesion site. This reduction makes neurons more susceptible to apoptosis or necrosis, decreases the ability of axonal growth, and affects the structure of synapses and the reconstruction of neural networks. Moreover, a variety of injury factors post‐TBI collectively stimulate and promote the proliferation and differentiation of neural stem cells (NSCs), thereby initiating endogenous neuroprotective and repair mechanisms.^[^ ^9^ ^]^ However, under the influence of NGF depletion at the injury site, NSCs rarely differentiate into neurons. Instead, they primarily differentiate into astrocytes, which form glial scars at the injury site. These glial scars act as physical and chemical barriers to axonal regeneration of neurons, further inhibiting synapse formation and neural circuit reconstruction. In response to the depletion of endogenous NGF at the injury site following TBI, the exogenous delivery of NGF becomes particularly crucial.

In early clinical trials, recombinant human NGF has been developed and typically administered to TBI patients via local routes. However, NGF's high molecular weight, complex advanced structure, hydrophilic property, and short half‐life generally prevent it from penetrating the blood‐brain barrier (BBB), blood‐cerebrospinal fluid barrier, and biological membrane barrier, greatly limiting its clinical application in the treatment of TBI.^[^ ^10^ ^]^ Despite early clinical failures, recombinant NGF's potential in TBI treatment is still worthy of further study.^[^ ^11^ ^]^ Thus, designing a targeted delivery system that efficiently protects NGF bioactivity, facilitates its delivery across the BBB, and promotes its accumulation in the lesion area holds great clinical significance.

Currently, researchers primarily utilize stem cell therapy,^[^ ^12^, ^13^ ^]^ hydrogels,^[^ ^14^, ^15^, ^16^ ^]^ viral vectors,^[^ ^17^ ^]^ and nanotechnology,^[^ ^18^, ^19^ ^]^ in an attempt to deliver biomacromolecules like NGF to promote neural regeneration and repair following TBI. Although these technologies have achieved certain progress in the exogenous delivery of NGF, they still face numerous challenges in the process of clinical translation. Stem cell therapy and hydrogel materials are typically involved in situ transplantation for drug delivery, and invasive surgeries might cause additional brain damage.^[^ ^12^, ^20^ ^]^ Additionally, issues such as immune rejection, tumorigenic risk, and low survival rate of in situ transplantation associated with stem cell therapy all restrict its clinical translation.^[^ ^21^ ^]^ In clinical applications, hydrogel materials need to address issues related to their biodegradability, mechanical properties, and long‐term stability.^[^ ^22^, ^23^ ^]^ Additionally, viral vectors have been criticized for being unsafe since the absence of transcriptional control.^[^ ^24^ ^]^ Nanotechnology‐based intravenous NGF delivery has also been explored, but brain delivery efficiency remains suboptimal.^[^ ^25^ ^]^

To facilitate the delivery of NGF to the brain, we have developed a biodegradable, biocompatible, and neurophilic biomimetic lipoprotein for nerve regeneration. Specifically, hyaluronic acid (HA) and protamine (PRTM) self‐assembled with NGF to form a slightly cationic core (Nc), which was subsequently wrapped by ApoE3‐reconstituted high‐density lipoprotein (rHDL) to generate Nc‐rHDL via electrostatic interactions. As previously reported, HA and PRTM, as extracellular and cytoplasmic matrices, can constrain the molecular mobility of protein cargos, diminish conformational changes, and thereby hinder their aggregation, maintaining thermodynamic stability and biological activity.^[^ ^26^ ^]^ ApoE3‐based rHDL simulates the role of the natural high‐density lipoprotein in transporting nutrients in the brain and crosses the BBB through low‐density lipoprotein receptor (LDLR) and LDLR‐related protein 1 (LRP1).^[^ ^27^ ^]^ To confer neurotropism, Nc‐rHDL was further grafted with a neurotropic peptide (αRDP) using a post‐insertion method. The αRDP peptide is a fusion peptide formed by combining rabies virus glycoprotein‐derived peptide (RDP) and an α‐helix peptide (FAEKFKEAVKDYFAKFWD). The α‐helix peptide mimics the lipid‐binding motif of apolipoprotein A‐I in binding to lipid membranes.^[^ ^28^, ^29^ ^]^ As a short peptide with 29 amino acids, RDP inherits the ability of rabies virus glycoproteins to bind specifically to the nicotinic acetylcholine receptors (nAchRs), which are widely expressed in the central nervous system (CNS) and on luminal brain capillary endothelium.^[^ ^30^ ^]^ Facilitated by ApoE3 and αRDP, the encapsulated NGF could first cross the BBB. Then, the acute injury could disrupt local vasculature and induce a decrease in pH, where the encapsulated NGF could be extravasated from the disorganized blood vessels and accumulated at the injury site, releasing NGF for neural regeneration.

Herein, we developed a neurophilic biomimetic lipoprotein loaded with NGF, designated as Nc‐rHDL@P, and systematically conducted its comprehensive characterization. Using an in vitro BBB model and controlled cortical impact (CCI) model mice, it was confirmed that the neurophilic biomimetic lipoprotein efficiently crossed the BBB and delivered NGF to damaged neurons. The efficient delivery of NGF by the neurophilic biomimetic lipoprotein has been shown to enhance the survival of damaged neurons and promote the differentiation of PC12 cells, human neural stem cells (hNSCs), and Schwann cells in vitro. The in vivo functional experiments conducted on CCI model mice confirmed that Nc‐rHDL@P enhanced motor and spatial learning and memory by promoting endogenous nerve regeneration. Collectively, we have successfully constructed a neurophilic biomimetic lipoprotein that promoted nerve repair and regeneration through targeted delivery of NGF and ultimately stimulated neurological function recovery. We envisage that the neurophilic biomimetic lipoprotein may unlock new therapeutic avenues in the treatment of TBI patients.

## 2 Results and Discussion

### 2.1 Preparation and Characterization of Nc‐rHDL@P

**Figure 1:**
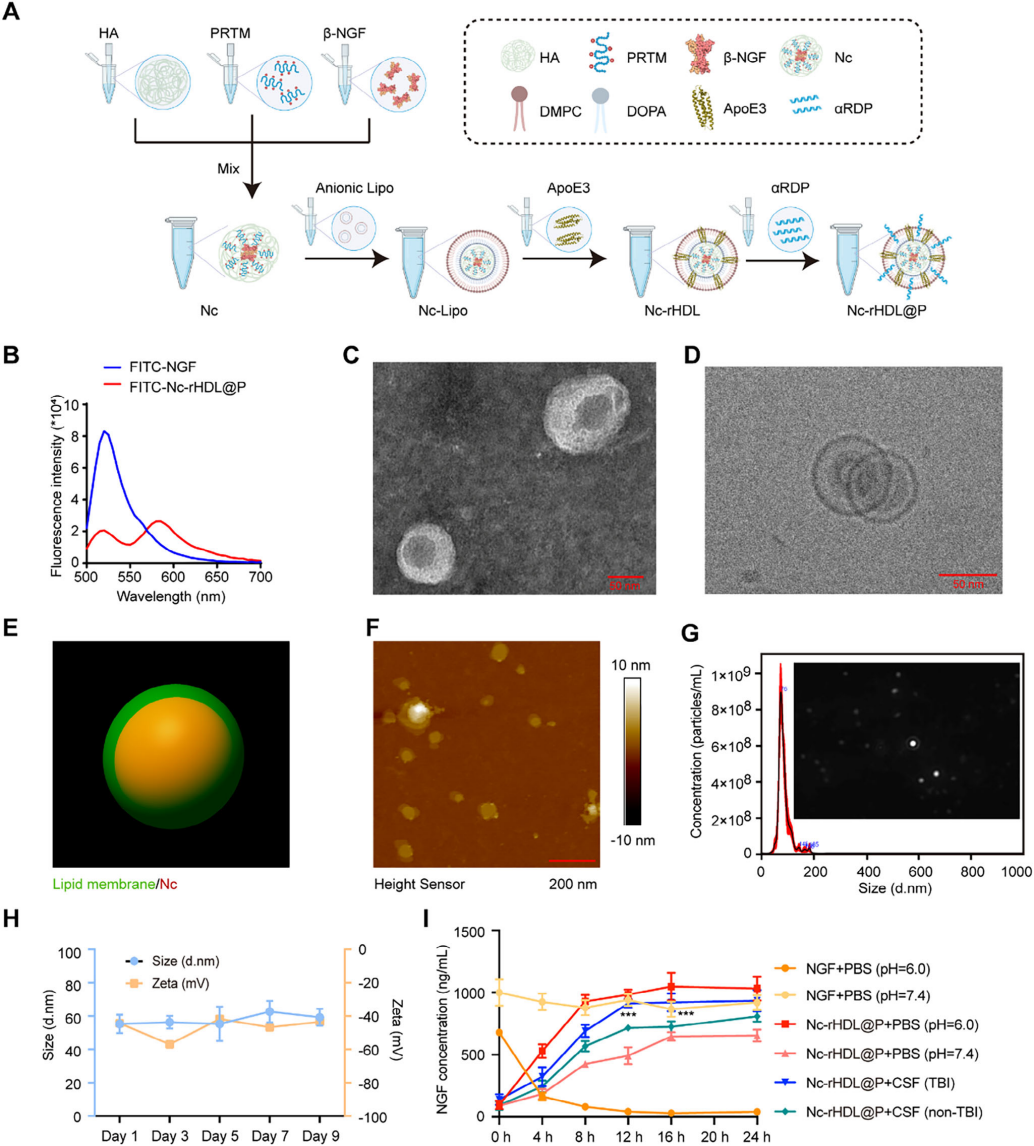
Preparation and characterization of Nc‐rHDL@P. A) The schematic illustration of self‐assembled Nc‐rHDL@P created in BioRender. Weng, W. (2025) (https://BioRender.com/25xnrv1). B) The characterization of Nc‐rHDL@P by FRET. FITC was used to label NGF, and RBITC to label the lipid membrane of Nc‐rHDL@P. Free FITC‐labeled NGF served as a control, and the concentration of NGF was 10 µg mL^−1^. The excitation and emission wavelengths were 470 nm and 500–700 nm, respectively. C) The morphology of Nc‐rHDL@P under TEM. Scale bar, 50 nm. D) The morphology of Nc‐rHDL@P under cryo‐TEM. Scale bar, 50 nm. E) Structure of Nc‐rHDL@P reconstructed with iMaris 3D. Alexa Fluor 647 was used to label NGF, and DiI to label the lipid membrane of Nc‐rHDL@P. F) The topographic image of Nc‐rHDL@P analyzed by AFM. Scale bar, 200 nm. G) Particle size distribution and concentration measured by NanoSight NS300. H) Particle size and zeta potential of Nc‐rHDL@P measured by DLS. I) In vitro simulated drug release of Nc‐rHDL@P in PBS (pH = 6.0), PBS (pH = 7.4), CSF of nontraumatic patients, and CSF of TBI patients. Data represent mean ± SD, *n* = 3. ^***^ *p* < 0.001 indicated that there was a significant difference between Nc‐rHDL@P+CSF (TBI) and Nc‐rHDL@P+CSF (non‐TBI). Two‐way ANOVA with Bonferroni's multiple comparisons was used.

As shown in **Figure** **1**A, a self‐assembly procedure was used to prepare Nc‐rHDL@P. First, we used PRTM and HA from the cell matrix component to create an NGF‐load core (Nc). HA is an anionic, nonsulfated glycosaminoglycan found throughout the body's connective tissue.^[^ ^31^ ^]^ PRTM is a naturally existing arginine‐rich cationic polypeptide that is biocompatible, biodegradable, and nontoxic.^[^ ^32^ ^]^ In nature, the positively charged PRTM binds negatively charged DNA during spermatogenesis and protects DNA against degradation.^[^ ^33^ ^]^ Here, we mimicked the in vivo self‐assembly mechanism to incorporate PRTM and HA as core carrier excipients for biologically active NGF. Second, anionic liposomes consisting of 1,2‐dimyristoyl‐*sn*‐glycero‐3‐phosphocholine (DMPC) and 1,2‐dioleoyl‐*sn*‐glycero‐3‐phosphate (DOPA) were prepared by thin film hydration followed by a probe ultrasound. Additionally, the above anionic liposomes were applied to coat the Nc via charge‐charge interaction to form the Nc‐Lipo (Table S1, Supporting Information). Third, Nc‐Lipo was subsequently incubated with ApoE3 and αRDP to yield Nc‐rHDL and Nc‐rHDL@P (Figure 1A). The empty biomimetic lipoprotein devoid of NGF was designated as c‐rHDL@P.

To investigate the formation of the nano‐formulation, fluorescence resonance energy transfer (FRET) was conducted to confirm the effective assembly of NGF with c‐rHDL@P (Figure 1B). FRET is a powerful technique used to study the interaction between two molecules by measuring the energy transfer between a donor and an acceptor fluorophore.^[^ ^34^ ^]^ In this study, NGF was labeled with a donor fluorophore (FITC), while c‐rHDL@P was labeled with an acceptor fluorophore (RBITC). The efficient assembly of NGF with c‐rHDL@P was confirmed through the observation of donor fluorescence quenching and acceptor fluorescence enhancement, indicating the close proximity and effective interaction between the two components (Figure 1B). Successful assembly was crucial for ensuring the stability and functionality of the nano‐formulation, which is essential for its therapeutic application in delivering NGF to target sites in the brain. Transmission electron microscopy (TEM) and cryo‐transmission electron microscopy (cryo‐TEM) analysis further confirmed that the neurophilic biomimetic lipoprotein successfully encapsulated the Nc, forming a core‐shell structure (Figure 1C,D). The TEM images of Nc‐Lipo and Nc‐rHDL further demonstrated the precursor and evolution process of this core‐shell structure (Figure S1A,B, Supporting Information). Nc‐Lipo also exhibited a core‐shell structure, but it was loose and uneven in shape, size, and distribution (Figure S1A, Supporting Information). Upon co‐incubation with ApoE3, the structure of Nc‐rHDL became more compact and the core‐shell structure more prominent (Figure S1B, Supporting Information). The above results suggested that after incubation with Nc‐Lipo, ApoE3's and ApoAI's α‐helix structure not only reduced the aggregation of Nc‐Lipo by interacting with the phospholipid bilayer of the liposome, but also optimized the lipid distribution within the liposome, thereby improving the compactness and uniformity. Moreover, we used DeltaVision OMX SR super‐resolution imaging and 3D reconstruction to further characterize the core‐shell structure. As shown in Figure 1E, the Alexa Fluor 647‐labeled NGF appeared yellow due to its encapsulation within the DiI‐labeled lipid membrane of the c‐rHDL@P. Also, an atomic force microscope (AFM) revealed that Nc‐rHDL@P consisted of core and surface lipids with a diameter of 50 nm (Figure 1F; Figure S1E, Supporting Information). By contrast, the majority of Nc tended to aggregate into particles of diverse sizes, exhibiting considerable heterogeneity (Figure S1C, Supporting Information). Empty liposome (Lipo) mainly presented hollow vesicle structures with varying sizes (Figure S1D). The above results suggested that Nc, Lipo, ApoE3, and αRDP can interact to form a more compact, smaller, and uniform Nc‐rHDL@P assembly through electrostatic interactions and α‐helix structural motif interactions.

Using Nanosight NS300, the concentration of Nc‐Lipo, Nc‐rHDL, and Nc‐rHDL@P was 1.28 × 10^9^ ± 9.35 × 10^7^, 2.26 × 10^9^ ± 3.42 × 10^8^, and 2.80 × 10^10^ ± 2.14 × 10^9^ particles per mL, respectively (Figure 1G; Figure S1G,H, Supporting Information). Using dynamic light scattering (DLS) measurement to detect the particle size, zeta potential, and stability of Nc‐rHDL@P. The results indicated that the particle size of Nc‐rHDL@P remained consistently within the range of 55–65 nm (Figure 1H). Meanwhile, the zeta potential was stably maintained between −40 and −60 mV (Figure 1H). When stored at 4 °C for nine days, the particle size and zeta potential exhibited minimal fluctuations, indicating good stability of Nc‐rHDL@P (Figure 1H). A good uniformity was also confirmed by Nc‐rHDL@P with polydispersity index (PDI) as low as 0.16–0.26 (Figure S1F, Supporting Information). Based on the experimental results obtained using Alexa Fluor 647‐labeled NGF as the fluorescent indicator, it was determined that a significant portion of NGF (the encapsulation efficiency reached an impressive 86.18%) was successfully encapsulated within the neurophilic biomimetic lipoprotein. This high encapsulation efficiency is crucial for ensuring the targeted delivery and sustained release of NGF in applications related to neural repair.

The pH value of lesion tissue after TBI usually decreases, and this acidic environment has a negative impact on the survival and functional recovery of nerve cells.^[^ ^35^ ^]^ However, it may promote the degradation of lipoproteins and drug release.^[^ ^36^ ^]^ Nc‐rHDL@P's core excipient, HA, exhibits a high swelling rate under acidic conditions because its large molecular chains have more free volume, which allows it to absorb more water.^[^ ^37^ ^]^ The swelling of Nc‐rHDL@P's core can promote NGF release when the volume of the core increases. For this research, we used PBS with different pH values, along with cerebrospinal fluid (CSF) from clinical TBI patients, to simulate an acidic microenvironment and study NGF's release kinetics in Nc‐rHDL@P. According to Figure 1I, the concentration of NGF rapidly decreased under acidic conditions, while under normal physiological pH conditions the rate of decrease significantly slowed down. Specifically, NGF suffered extensive loss of its intact form within just 4 h of exposure to acidic conditions (Figure 1I). Further analysis indicated that the rapid decrease in free NGF concentration under acidic conditions was due to aggregation (Figure S2, Supporting Information). As the exposure time extended to 24 h, cumulative NGF depletion reached 95.87%, underscoring the marked vulnerability of NGF to prolonged acid exposure, which induced severe structural instability (Figure 1I). These findings indicated the importance of protecting NGF from acidic environments to maintain its structural integrity and therapeutic efficacy, especially in applications where NGF was used for neural repair and other biomedical purposes. Therefore, it was particularly important to use lipoproteins to protect NGF. When NGF was loaded into the c‐rHDL@P structure, it was shielded from the harsh conditions typically found in acidic environments, such as those present in endosomes or lysosomes within cells, or in certain pathological tissues. The c‐rHDL@P acted as a robust barrier, maintaining the structural integrity and biological activity of NGF for a significantly longer period compared to free NGF exposed directly to acidic conditions (Figure 1I). Moreover, compared with normal physiological pH environments, Nc‐rHDL@P achieved faster and more efficient drug release in acidic conditions (Figure 1I). The enhanced release rate in acidic conditions suggested that Nc‐rHDL@P was highly responsive to pH changes. Given that many pathological environments often exhibited a lower pH than normal tissues,^[^ ^38^ ^]^ this pH‐sensitivity facilitated a more controlled and site‐specific delivery of NGF. It ensured that NGF was predominantly released in the target environment where it is most needed, thereby enhancing therapeutic efficacy while minimizing potential side effects in non‐target tissues. Consistent with the above findings, when CSF from patients with TBI was introduced, it triggered a more rapid release of NGF from the Nc‐rHDL@P compared to the release observed in the presence of CSF from non‐traumatic patients (Figure 1I). Altogether, these results demonstrated that the encapsulation of NGF using c‐rHDL@P improved its stability and enabled the efficient release of NGF in the lesion's microenvironment.

### 2.2 Nc‐rHDL@P Promoted Nerve Cell Survival, Differentiation, and Neurotrophic Factor Expression

**Figure 2:**
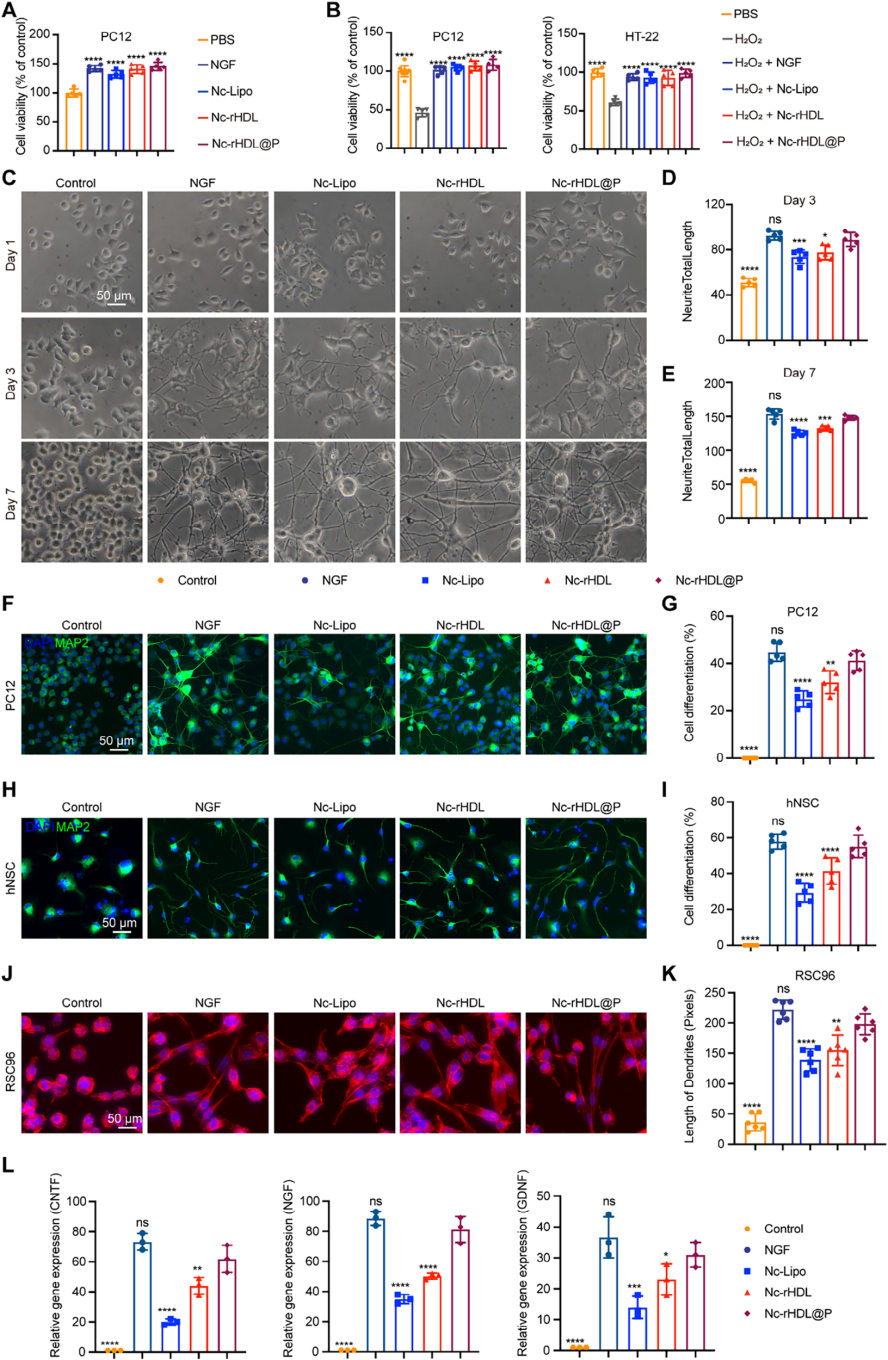
Nc‐rHDL@P promoted nerve cell survival, differentiation, and neurotrophic factor expression. A) Cell viability of PC12 cells after treatment with free NGF and NGF formulations, as measured by CCK‐8 assays. B) Cell viability of PC12 and HT22 cells after H_2_O_2_‐induced oxidative damage and treatment with free NGF and NGF formulations. C) Light microscopic images of PC12 cells cultured over 7 days and treated with free NGF and NGF formulations at the NGF concentration of 100 ng mL^−1^. The control group was treated without NGF supplements. D,E) Neurite total length of PC12 cells after 3‐day D) and 7‐day E) treatments. F) Representative fluorescent images of PC12 cells following 7‐day treatment with free NGF and NGF formulations at the NGF concentration of 100 ng mL^−1^. Neurite outgrowth was visualized by MAP2 staining (green). Scale bar, 50 µm. G) The cell differentiation percentage of PC12 cells with neurite outgrowth was estimated for each group after 7‐day treatment. H) Fluorescent images of hNSC following 7‐day treatment with free NGF and NGF formulations at the NGF concentration of 100 ng mL^−1^. Scale bar, 50 µm. I) The cell differentiation percentage of hNSC with neurite outgrowth was estimated for each group after 7‐day treatment. J) Fluorescent images of RSC96 cells following 3‐day treatment with free NGF and NGF formulations at the NGF concentration of 100 ng mL^−1^. K) Length of dendrites in RSC96 cells after 3‐day treatment. L) Relative mRNA expression levels of neurotrophic genes (CNTF, NGF, and GDNF) in RSC96 cells after 3‐day treatment by RT–qPCR. Data represent mean ± SD, *n* ≥ 3. ^*^ *p* < 0.05, ^**^ *p* < 0.01, ^***^ *p* < 0.001, and ^****^ *p* < 0.0001. ns, not significant. One‐way ANOVA with Bonferroni's multiple comparisons was used.

After verifying that NGF was efficiently encapsulated by the neurophilic biomimetic lipoprotein and demonstrating its good stability as well as effective drug release performance in the lesion microenvironment, we proceeded to further evaluate the biological activity of the released NGF. This step is crucial to ensure that the encapsulated NGF not only remains stable and releases effectively but also retains its full biological functionality once released. We conducted a series of in vitro experiments to assess the biological activity of the released NGF, focusing on its ability to promote nerve cell survival, differentiation, neurite outgrowth, and support overall neural repair processes. We first tested the effect of NGF formulations on cell viability using the cell counting kit‐8 (CCK‐8) assay and found that both free NGF and NGF preparations promoted the proliferation of PC12 cells without adversely affecting cell viability (**Figure** **2**A). Moreover, we used a hydrogen peroxide‐induced cell damage model to simulate the oxidative stress microenvironment after brain injury.^[^ ^39^ ^]^ Based on the hydrogen peroxide injury concentration screening experiment, cell viability can be reduced by 50%–75% when 50 µm hydrogen peroxide causes damage for 2 h in PC12 cells and 200 µm hydrogen peroxide causes injury for 2 h in HT‐22 cells (Figure S3, Supporting Information). Therefore, the above concentrations were used to construct the model of oxidative injury in nerve cells. As shown in Figure 2B, both free NGF and NGF preparations promoted the survival of PC12 and HT‐22 cells, with survival rates approaching the level of undamaged controls. Preparations containing NGF did not damage neuronal vitality and instead promoted their survival following injury.

As PC12 cells were widely used to examine whether the carrier loading affects the biological activity of NGF, free NGF, and NGF‐loaded formulations were incubated with PC12 cells for 7 days to observe the neurite outgrowth.^[^ ^40^ ^]^ Light microscopic images showed that both free NGF and various NGF‐loaded formulations effectively promoted the differentiation of PC12 cells, characterized by significant neurite outgrowth (Figure 2C–E). In contrast, untreated control PC12 cells maintained their rounded morphology and exhibited no neurite formation, regardless of whether they were observed after 1, 3, or 7 days (Figure 2C–E). Specifically, free NGF, Nc‐Lipo, Nc‐rHDL, and Nc‐rHDL@P all significantly increased neurite lengths after 7 days of culture by 183.25%, 110.02%, 128.13%, and 176.14%, respectively (Figure 2E). As compared to Nc‐Lipo and Nc‐rHDL, Nc‐rHDL@P significantly accelerated neurite outgrowth in PC12 cells after 3 or 7 days of incubation, but with no significant differences compared with the free NGF group, indicating that c‐rHDL@P encapsulation did not affect NGF's biological activity (Figure 2D,E).

To further assess the impact of various NGF formulations on PC12 cell differentiation, microtubule‐associated protein 2 (MAP2) immunostaining was performed after a 7‐day culture period. This technique allows for the visualization and quantification of differentiated PC12 cells by specifically labeling MAP2, which is characteristic of neurite outgrowth and neuronal differentiation.^[^ ^41^ ^]^ By comparing the staining intensity and patterns across different NGF formulations, we were able to determine the percentage of PC12 cells that had successfully differentiated into neuron‐like cells with extended neurites. As shown in Figure 2F,G, the differentiation ratio of PC12 cells induced by Nc‐rHDL@P was 41.22%, similar to that induced by free NGF (44.82%), and significantly higher than that of Nc‐Lipo‐treated cells (24.95%) and Nc‐rHDL‐treated cells (32.10%). Further, high‐content analysis (HCS) was used to quantify neurite length and branching complexity of MAP2+ cells differentiated after different treatments. It was found that in the control group, the neurite total length was 31.27 ± 3.72 µm, and the branch point total count was 1.34 ± 0.21 (Figure S4A, Supporting Information). In contrast, the various NGF formulations—free NGF, Nc‐Lipo, Nc‐rHDL, and Nc‐rHDL@P—all significantly enhanced both the neurite total length and the branch point total count. Specifically, the neurite total length increased by 340.36%, 272.05%, 300.20%, and 333.10%, respectively, while the branch point total count rose by 158.96%, 33.58%, 64.85%, and 132.84%, respectively (Figure S4A, Supporting Information). Meanwhile, in the realm of neurobiological research, the differentiation of hNSCs into neurons has emerged as a groundbreaking and innovative evaluation system for various NGF formulations. The hNSCs were incubated in the induction medium with free NGF, Nc‐Lipo, Nc‐rHDL, or Nc‐rHDL@P for 7 days and then stained by immunohistochemistry with MAP2. Nc‐rHDL@P demonstrated the best ability to promote hNSC differentiation, and its effect was comparable to that of free NGF, as shown in Figure 2H. The percentage of neuronal differentiation in hNSCs treated with free NGF, Nc‐Lipo, Nc‐rHDL, and Nc‐rHDL@P was 57.93%, 29.26%, 41.37%, and 55.18%, respectively (Figure 2I). In addition, HCS showed that, compared with Nc‐Lipo and Nc‐rHDL, Nc‐rHDL@P significantly increased neurite total length and the number of branch points, comparable to free NGF treatment (Figure S4B, Supporting Information). The above results indicated that Nc‐rHDL@P not only promoted a high percentage of neuronal differentiation but also supported the development of functional neuronal networks. This comprehensive evaluation highlighted the potential of Nc‐rHDL@P as an effective and advanced NGF delivery formulation for applications in neural regeneration and therapeutic interventions.

In addition to hNSCs mentioned earlier, RSC96 cells, which are a well‐established rat Schwann cell line, play a crucial role in neuroscience research. One of the key applications of RSC96 cells is in the detection of the biological activity of NGF. RSC96 cells respond to NGF stimulation by undergoing morphological changes and expressing specific markers, making them an excellent model for assessing the efficacy of NGF formulations.^[^ ^42^ ^]^ Here, free NGF and the different NGF formulations were supplemented in the culture medium for the RSC96 cell culture. By analyzing cell morphology with phalloidin staining during the culture process, the Nc‐rHDL@P group showed greater differentiation than other NGF formulations (Figure 2J). Data analysis showed that the dendrite length increased by 516.67% for the free NGF group compared to the untreated control group, 286.11% for Nc‐Lipo group, and 330.56% for the Nc‐rHDL group, as well as 450% for the Nc‐rHDL@P group, which was significantly higher than the dendrite length of the Nc‐Lipo and Nc‐rHDL groups and close to that of the group treated with free NGF (Figure 2K). Moreover, RSC96 cells, as neurotrophic cells, have the remarkable ability to secrete a wide array of neurotrophic factors, including NGF, glial cell‐derived neurotrophic factor (GDNF), and ciliary neurotrophic factor (CNTF), all of which are critical to neurons' survival, regeneration, and health. Therefore, RT‐qPCR was used to measure the expression of neurotrophic factors in RSC96 cells treated with different formulations of NGF. As shown in Figure 2L, treatment with free NGF resulted in high levels of CNTF, NGF, and GDNF gene expression. In addition to this, the neurotrophin gene expression of Nc‐rHDL@P treated cells was significantly higher after 3 days of culture compared to Nc‐Lipo and Nc‐rHDL‐treated cells. Further analysis suggests that the αRDP peptide on the surface of Nc‐rHDL@P may enhance the interaction between the loaded NGF and the neuronal membrane. Moreover, there was no significant difference between the cells of the Nc‐rHDL@P treatment group and those of the free NGF treatment group when it came to the gene expression of neurotrophic factors, indicating that Nc‐rHDL@P protected NGF biological activity and promoted its neuroprotective function similar to free NGF. Based on these results, it was evident that Nc‐rHDL@P promoted the survival, differentiation, and neurotrophic factor‐related gene expression of neurotrophic cells, which was conducive to nerve repair and regeneration.

### 2.3 Nc‐rHDL@P Efficiently Targeted Lesion Sites and Achieved Neuron‐Targeted Delivery of NGF in CCI Mice

**Figure 3:**
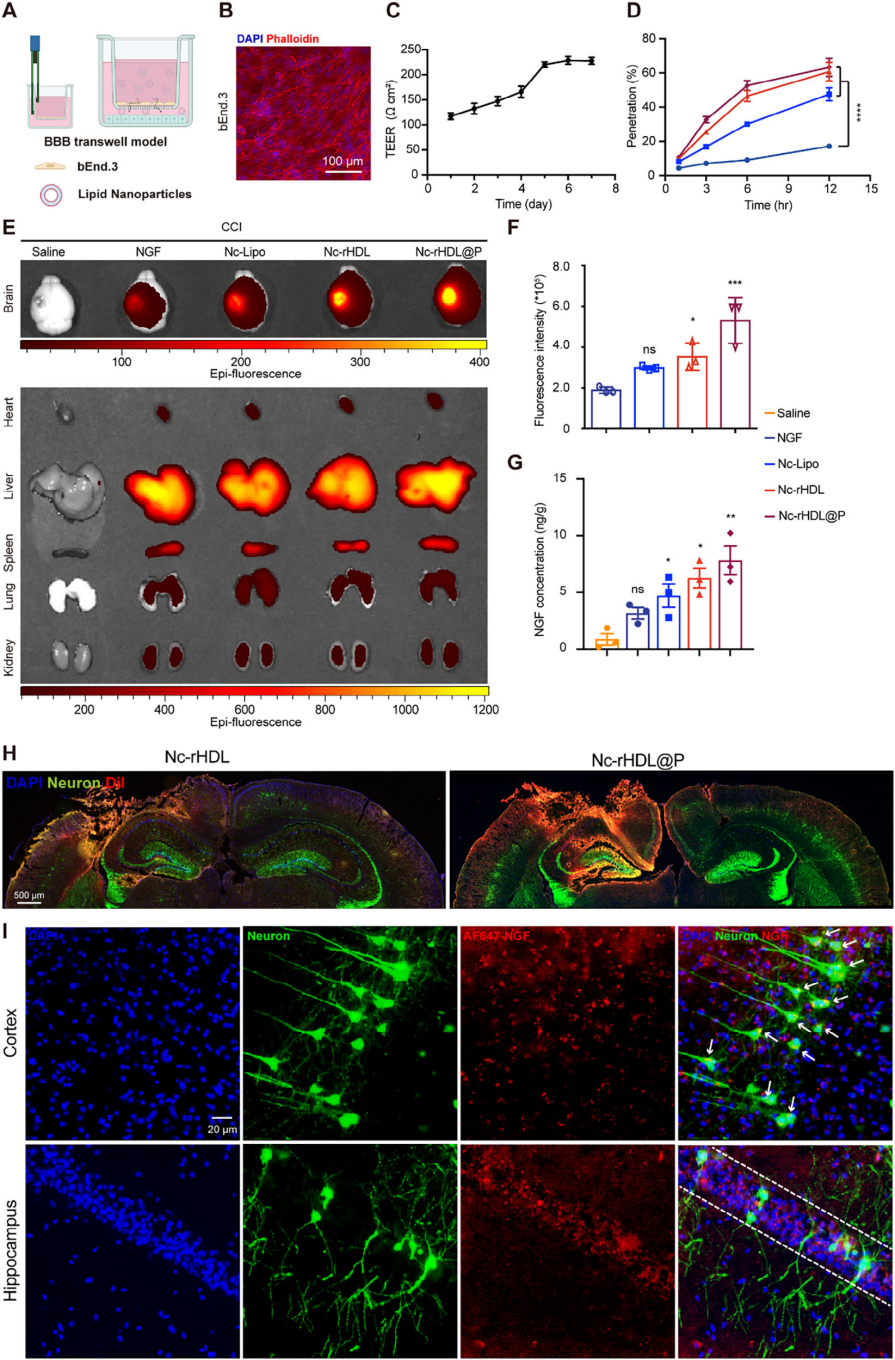
Nc‐rHDL@P efficiently targeted lesion sites and achieved neuron‐targeted delivery of NGF in CCI mice. A) The schematic illustration of the in vitro BBB model measuring the penetration of Nc‐rHDL@P created in BioRender. Weng, W. (2025) (https://BioRender.com/25xnrv1). The concentration of Alexa Fluor 647‐labeled NGF for formulations was 100 µg mL^−1^. B) Cell monolayer of bEnd.3 cells after 7‐day cell culture. The actin and nuclei were stained with Rhodamine‐Phalloidin and DAPI, respectively. Scale bar, 100 µm. C) TEER value of the in vitro BBB model from the first day to the seventh day. D) Penetration percentage of free NGF and NGF formulations through the in vitro BBB model. Calculation of penetration percentage was performed by normalizing the fraction of formulations that penetrated blank filter inserts. E) Biodistribution of Alexa Fluor 647‐labeled free NGF and NGF formulations at 4 h after administration at the NGF dose of 2.5 mg kg^−1^ in CCI model mice. F) Semi‐quantitative analysis of the brain distribution of Alexa Fluor 647‐labeled free NGF and NGF formulations. G) Brain NGF concentration at 4 h after administration at the NGF dose of 2.5 mg kg^−1^ in CCI model mice by ELISA kit. H) Brain distribution of DiI‐labeled NGF formulations at 4 h after administration at the DMPC dose of 5 mg kg^−1^ in Thy1‐GFP mice with CCI. Green: GFP‐labeled neuron. Scale bar, 500 µm. I) Brain distribution of Alexa Fluor 647‐labeled Nc‐rHDL@P at 4 h after administration at the NGF dose of 2.5 mg kg^−1^ in Thy1‐GFP mice with CCI. Scale bar, 20 µm. The white arrows indicated the colocalization of Alexa Fluor 647‐labeled NGF with GFP‐labeled pyramidal neurons in the cortex. The white dashed lines indicated the accumulation of Alexa Fluor 647‐labeled NGF in the hippocampus. Data represent mean ± SD, *n* ≥ 3. ^*^ *p* < 0.05, ^**^ *p* < 0.01, ^***^ *p* < 0.001, and ^****^ *p* < 0.0001. ns, not significant. One‐way ANOVA with Bonferroni's multiple comparisons test was used for (F) and (G). Two‐way ANOVA with Bonferroni's multiple comparisons test was used for (D).

Low BBB permeability affects drug delivery to the brain, particularly for macromolecular protein therapeutics.^[^ ^43^ ^]^ Despite changes to BBB integrity following TBI, the increased permeability is often non‐specific and unstable, making its use challenging.^[^ ^44^ ^]^ After verifying that the encapsulation within the neurophilic biomimetic lipoprotein did not compromise the biological activity of NGF, we further assessed whether such encapsulation could facilitate NGF's traversal of the BBB and its targeted distribution to brain injury lesions. To investigate the BBB penetration efficiency of Nc‐rHDL@P, we first explored the uptake efficiency of NGF by bEnd.3 cells, a mouse brain endothelial cell line. Compared with free Alexa Fluor 647‐labeled NGF, the neurophilic biomimetic lipoprotein significantly improved the cellular uptake efficiency of NGF by bEnd.3 cells (Figure S5A, Supporting Information). Moreover, the endocytosis efficiency of bEnd.3 cells for Nc‐rHDL@P was 3.06 times that of free NGF, 1.67 times that of Nc‐Lipo, and 1.35 times that of Nc‐rHDL (Figure S5B, Supporting Information). This indicated that Nc‐rHDL@P could deliver NGF into the bEnd.3 cells more effectively, leveraging the core and rHDL encapsulation along with αRDP for enhanced delivery. Further, we established a transwell BBB model with bEnd.3 cells in the apical compartment to investigate the permeability and transport characteristics of various NGF formulations across the BBB. The bEnd.3 cells, a well‐characterized immortalized mouse brain endothelial cell line, were seeded in the apical compartment of the transwell system, which recapitulated the physiological environment of the BBB. This model allowed us to assess the ability of free NGF, Nc‐Lipo, Nc‐rHDL, and Nc‐rHDL@P to cross the endothelial barrier and reach the basal compartment, thereby providing insights into their potential for brain delivery (**Figure** **3**A). The formation of a robust BBB monolayer was confirmed using phalloidin staining, which highlighted filamentous actin and delineated cell morphology, indicating proper cell alignment and tight junction formation (Figure 3B). Additionally, the transendothelial electrical resistance (TEER) value exceeded 200  Ω·cm², a hallmark of a well‐formed BBB (Figure 3C). This high TEER value signified a significant reduction in paracellular permeability, ensuring that the monolayer effectively mimicked the barrier properties of the BBB. After the formation of the BBB monolayer, Alexa Fluor 647‐labeled NGF preparations were added to the apical compartment, and the filtrate from the basal compartment was collected at different time points for fluorescence intensity analysis. By normalizing the fraction of preparations that penetrated blank filter inserts, the percentage of NGF preparations that penetrated the monolayer was calculated. After incubation with the endothelial cell layer for 12 h, the permeation efficiencies of free NGF, Nc‐Lipo, Nc‐rHDL, and Nc‐rHDL@P for the in vitro BBB model were 17.09%, 47.58%, 60.80%, and 63.32%, respectively (Figure 3D). To verify the neurophilicity of Nc‐rHDL@P, BV‐2 and SH‐SY5Y cells were cultured in the basal compartment, and their uptake efficiency of NGF formulations introduced into the apical compartment was assessed. As shown in Figure S6 (Supporting Information), the endocytosis efficiency of BV‐2 cells for Nc‐rHDL@P was 1.32 times that of Nc‐rHDL, 1.43 times that of Nc‐Lipo, and 2.99 times that of free NGF. Similarly, the endocytosis efficiency of SH‐SY5Y cells for Nc‐rHDL@P was 1.09 times that of Nc‐rHDL, 1.37 times that of Nc‐Lipo, and 4.24 times that of free NGF (Figure S6B,C, Supporting Information). In addition to labeling NGF with Alexa Fluor 647, we also labeled the lipid membrane of c‐rHDL@P with DiI to further investigate the carrier's neurophilicity. Figure S7A,C (Supporting Information) showed that Nc‐Lipo, which lacked neurophilicity, was less distributed around the soma of primary neurons. In contrast, Nc‐rHDL assembled with ApoE3 was more extensively distributed around the soma and processes of primary neurons (Figure S7A, Supporting Information). Moreover, Nc‐rHDL@P formed by assembling Nc‐rHDL with αRAP peptide, exhibited the most efficient distribution around the soma and processes of primary neurons (Figure S7A,C, Supporting Information). The aforementioned results indicated that the assembly of ApoE3 to form rHDL and its subsequent assembly with αRAP peptide progressively enhanced the neurophilicity of the biomimetic nanocarrier, ultimately resulting in the formation of the neurophilic biomimetic lipoprotein c‐rHDL@P. The same conclusion was also confirmed in HT22 cells (Figure S7B,C, Supporting Information). The above results demonstrated that the encapsulation of NGF within c‐rHDL@P has been shown to significantly enhance its delivery across the BBB in vitro, facilitating its interaction with nerve cells, thereby promoting endocytosis. This action has the potential to improve the inflammatory microenvironment, promote neuronal survival, and ultimately enhance therapeutic efficacy for CNS disorders.

However, the persuasiveness of the in vitro BBB model is limited. The BBB penetration and neuron targeting efficiency of Nc‐rHDL@P were further explored in the CCI model mice using C57BL/6 mice and Thy1‐GFP transgene mice. Alexa Fluor 647 and DiR/DiI dyes were used to label NGF and the lipid membrane of c‐rHDL@P, respectively, to track their fate in vivo and intracerebral distribution. αRDP, a polypeptide derived from the rabies virus glycoprotein, is highly neurotropic and can bind to nAchRs in the CNS during crossing the BBB via receptor‐mediated transcytosis.^[^ ^45^ ^]^ It was therefore not surprising to discover that Alexa Fluor 647‐labeled Nc‐rHDL@P markedly increased the distribution of NGF in brain damage lesions in comparison to free NGF, Nc‐Lipo, and Nc‐rHDL (Figure 3E). Specifically, it was found that fluorescence intensity (Alexa Fluor 647‐labeled NGF) of Nc‐rHDL@P in the brain lesion of CCI mice was enhanced by 1.81, 0.78, and 0.50 times compared to that of free NGF, Nc‐Lipo, and Nc‐rHDL at 4 h after administration (Figure 3F). Besides using A647 to trace NGF, DiR was also used to trace the lipid shell of the neurophilic biomimetic lipoprotein, and we found the same phenomenon, that is, Nc‐rHDL@P enriched brain injury tissue more efficiently than other formulations, and the peak was obtained between 12 and 48 h after injection (Figures S8A, and S9, Supporting Information). Fluorescent quantitative statistical analysis showed that the fluorescence intensity in the brains of the Nc‐rHDL@P administration group increased by 57.50% compared with that in the Nc‐Lipo group and by 24.19% compared with that in the Nc‐rHDL group (Figure S8B, Supporting Information). There was no significant difference in the distribution of fluorescence intensity in the organs of CCI mice with the administration of different formulations (Figure S8C, Supporting Information). Efficient brain‐targeted distribution resulted in elevated NGF concentration in brain injury tissue. By using an enzyme‐linked immunosorbent assay (ELISA) kit, NGF concentrations in brain lesion tissues of mice treated with Nc‐rHDL@P increased by 147.15% when compared with those treated with free NGF, 65.8% when compared with Nc‐Lipo, and 24.76% when compared with Nc‐rHDL (Figure 3G). To more specifically localize Nc‐rHDL@P within brain injury tissue, C57BL/6 and Thy1 mice with GFP‐labeled neurons were used to construct the CCI model, and their brains were sectioned for targeted efficiency analysis. In comparison to Nc‐rHDL, Nc‐rHDL@P demonstrated enhanced distribution to the lesion site (Figure 3H). Specifically, while Nc‐rHDL was primarily localized in the cortex of the brain injury tissue, Nc‐rHDL@P not only penetrated the cortical regions but also achieved substantial enrichment in the hippocampal tissue on the injured side. This enrichment was particularly pronounced in the CA1, CA2, and dentate gyrus (DG) regions, highlighting the superior targeting capabilities of Nc‐rHDL@P. Owing to the incorporation of the αRDP functional group, Nc‐rHDL@P exhibited markedly elevated fluorescence signals within the injured brain, surpassing those of control formulations devoid of the αRDP modification (Figure S10, Supporting Information). The αRDP peptides, known for their neurotropic properties, facilitated the selective uptake and distribution of Nc‐rHDL@P in areas with high neuronal density, particularly in the cortex and hippocampus (Figure 3I; Figures S10, and S11, Supporting Information). This selective targeting not only increased the local concentration of NGF, thereby promoting neuronal survival and repair but also minimized the potential side effects associated with widespread distribution of NGF throughout the brain.^[^ ^46^, ^47^ ^]^ Collectively, owing to the bionic characteristics of rHDL and the high neuron‐binding affinity of αRDP, Nc‐rHDL@P efficiently crossed the BBB and trafficked to the local lesion to realize the neuron‐directed delivery of NGF, which was ready for further neuronal regeneration and repair.

### 2.4 Nc‐rHDL@P Improved the Inflammatory Microenvironment and Attenuated Neuropathological Damage in CCI Model Mice

**Figure 4:**
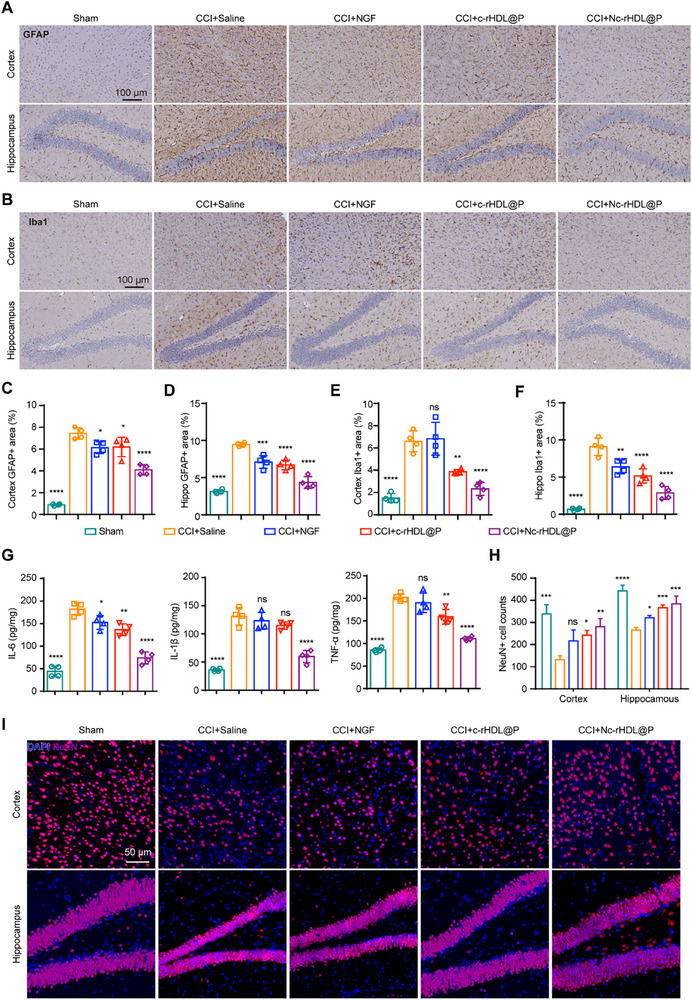
Nc‐rHDL@P improved the inflammatory microenvironment and attenuated neuropathological damage in the CCI model mice. A) GFAP immunostaining of the cortex and hippocampus, indicating astrogliosis in the CCI model mice after the treatment of free NGF or NGF formulations. Scale bar, 100 µm. B) Iba1 immunostaining of the cortex and hippocampus, indicating microglial activation in the CCI model mice after the treatment of free NGF or NGF formulations. Scale bar, 100 µm. C–F) Semi‐quantitative analysis of cortex GFAP+ area C), hippo GFAP+ area D), cortex Iba1+ area E), hippo Iba1+ area F). G) The expression levels of inflammatory factors in the CCI model mice after the treatment of free NGF or NGF formulations. H) Semi‐quantitative analysis of Neuron cell counts indicated by NeuN+ cells in the CCI model mice after the treatment of free NGF or NGF formulations. I) NeuN immunofluorescence of the cortex and hippocampus in CCI model mice after the treatment of free NGF and NGF formulations. Data represent mean ± SD, *n* = 4. ^*^ *p* < 0.05, ^**^ *p* < 0.01, ^***^ *p* < 0.001, and ^****^ *p* < 0.0001. ns, not significant. One‐way ANOVA with Bonferroni's multiple comparisons test was used.

The unfavorable inflammatory microenvironment after TBI is detrimental to the survival and synaptic plasticity of neurons.^[^ ^48^ ^]^ NGF not only supports neuronal survival through neurotrophic effects, but also exerts direct anti‐inflammatory effects by inhibiting inflammatory signaling pathways. After confirming the targeted distribution of Nc‐rHDL@P across the BBB to the brain injury site and its ability to promote neuronal survival, differentiation, and neurotrophic factor expression, we further evaluated through in vivo experiments whether Nc‐rHDL@P can improve the inflammatory microenvironment of injury lesions and their neuropathological damage. Upon brain injury, glia cells rapidly alter their morphology and release pro‐inflammatory cytokines such as tumor necrosis factor‐alpha (TNF‐α), interleukin‐1 beta (IL‐1β), and interleukin‐6 (IL‐6), thereby triggering an inflammatory response and promoting the formation of glial scars.^[^ ^49^ ^]^ The brain tissue was stained using markers for inflammatory cells, including astrocytes (GFAP—glial fibrillary acidic protein) and microglia (Iba‐1—ionized calcium‐binding adapter molecule 1). The level of glial scar tissue was monitored by immunostaining after treatment with different formulations. CCI induced significant glial scar formation, whereas Nc‐rHDL@P significantly alleviated it (**Figure** **4**A; Figure S12, Supporting Information). In CCI mice treated with Nc‐rHDL@P, a significant reduction in the number of Iba1+ microglia and GFAP+ astrocytes was observed in the ipsilateral hemispheric areas (Figure 4A,B; Figures S12, and S13, Supporting Information). Specifically, the proportion of GFAP+ astrocytes in the cortex dropped from 7.44% to 4.10%, and in the hippocampus, it fell from 9.46% to 4.34% (Figure 4C,D). Similarly, the percentage of Iba1+ microglia in the cortex decreased from 6.60% to 2.33%, and in the hippocampus, it decreased from 9.09% to 2.88% (Figure 4E,F). These effects were also supported by the decreased expression of the proinflammatory cytokines. As shown in Figure 4G, Nc‐rHDL@P significantly reduced the levels of pro‐inflammatory cytokines IL‐6, IL‐1β, and TNF‐α. In CCI mice treated with Nc‐rHDL@P, the expression of IL‐6 decreased from 181.97 to 74.03 pg mg^−1^, the expression of IL‐1β decreased from 131.29 to 59.73 pg mg^−1^, and that of TNF‐α decreased from 201.93 to 110.83 pg mg^−1^, respectively (Figure 4G). Together with NGF, the ameliorated inflammatory microenvironment at the brain injury site further promoted neuronal survival (Figure 4H,I). Following CCI, a significant reduction in the percentage of NeuN‐positive cells was observed in the cortex and hippocampus. Specifically, in the cortex, the proportion of NeuN‐positive cells decreased by 60.99%, while in the hippocampus, the reduction was 39.89% (Figure 4H). Treatment with Nc‐rHDL@P restored the percentage of NeuN‐positive cells to 82.89% in cortex, and 86.88% in the hippocampus, respectively (Figure 4H). The effect of Nc‐rHDL@P in promoting the survival of neurons in the cortex and hippocampus was also confirmed by Nissl staining (Figure S14A–F, Supporting Information). As shown in Figure S14B–F (Supporting Information), Nc‐rHDL@P‐treated CCI mice achieved a 181.31% increase in the neuronal count in the cortex compared with that in the saline‐treated CCI mice, while free NGF‐treated CCI mice only achieved a 16.17% increase (no significant difference), and c‐rHDL‐treated CCI mice achieved a 112.02% increase. Consistent with the above results, CCI model mice treated with Nc‐rHDL@P had the highest survival rate of neurons in the CA1, CA2, CA3, and DG regions of the hippocampus, approaching that of sham mice (Figure S14A–F, Supporting Information). According to the above results, free NGF alone might be ineffective in enhancing neuronal recovery with limited brain entry. The neuroprotective effect observed in the empty vector group was likely to be primarily attributed to the inherent properties of the rHDL shell. Specifically, the rHDL shell is known to possess potent anti‐inflammatory and antioxidant capabilities, which play crucial roles in mitigating the detrimental effects of neuroinflammation and oxidative stress often associated with various neurological disorders.^[^ ^50^, ^51^, ^52^ ^]^ In addition to evaluating neuronal survival through Nissl staining, we further employed Golgi‐Cox staining to meticulously examine the morphology of neurons and the alterations in dendritic spines. This comprehensive approach allowed us to gain deeper insights into the structural integrity and functional capacity of neurons, as well as the intricate changes occurring at the synaptic level. Compared to the saline‐treated CCI mice, the density of dendritic spines in Nc‐rHDL@P‐treated CCI mice was significantly increased by 120.43%, reaching levels comparable to those observed in sham mice (Figure S14G, Supporting Information). In contrast, free NGF and c‐rHDL@P treatments showed relatively modest effects, with increases of 35.71% and 50%, respectively (Figure S14G, Supporting Information). Further, we constructed the CCI model using Thy1‐GFP transgenic mice in which pyramidal neurons express green fluorescence. After different treatments, it was observed that the cortical neuronal survival in the Nc‐rHDL@P treatment group was increased, and the neuronal processes were better maintained compared with those in the CCI model mice of other treatment groups (Figure S15, Supporting Information). Corresponding to the above results, the expression of growth‐associated protein 43 (GAP43) in the hippocampus of CCI model mice treated with Nc‐rHDL@P was increased compared with that of CCI model mice in other treatment groups (Figure S16, Supporting Information). GAP43 is a key protein involved in axonal growth and regeneration. The increased expression of GAP43 in the hippocampus after Nc‐rHDL@P treatment signified enhanced neuronal survival and regeneration, improved synaptic plasticity, and neuroprotection. To further examine neuronal differentiation in the hippocampus of CCI mice, doublecortin (DCX) immunofluorescence was performed. DCX is currently recognized as a specific marker for immature neurons and is primarily used to detect newly generated neurons during neurogenesis. It is considered the “gold standard” for tracking the quantity and differentiation status of newborn neurons. As illustrated in Figure S17 (Supporting Information), Nc‐rHDL@P administration robustly up‐regulated DCX expression within the DG, restoring it to levels virtually indistinguishable from those observed in the sham group. This finding underscored the pronounced capacity of Nc‐rHDL@P therapy to drive post‐injury neuroregeneration in the CCI mouse model. As a result of the findings mentioned above, the empty vector c‐rHDL@P, constructed on the foundation of rHDL, was capable of enhancing the inflammatory microenvironment and promoting neuronal survival to a limited extent. By contrast, NGF loaded into c‐rHDL@P achieved a superior synergistic effect.

### 2.5 Nc‐rHDL@P Improved Sensorimotor Function and Rescued Cognitive Deficits in CCI Model Mice

**Figure 5:**
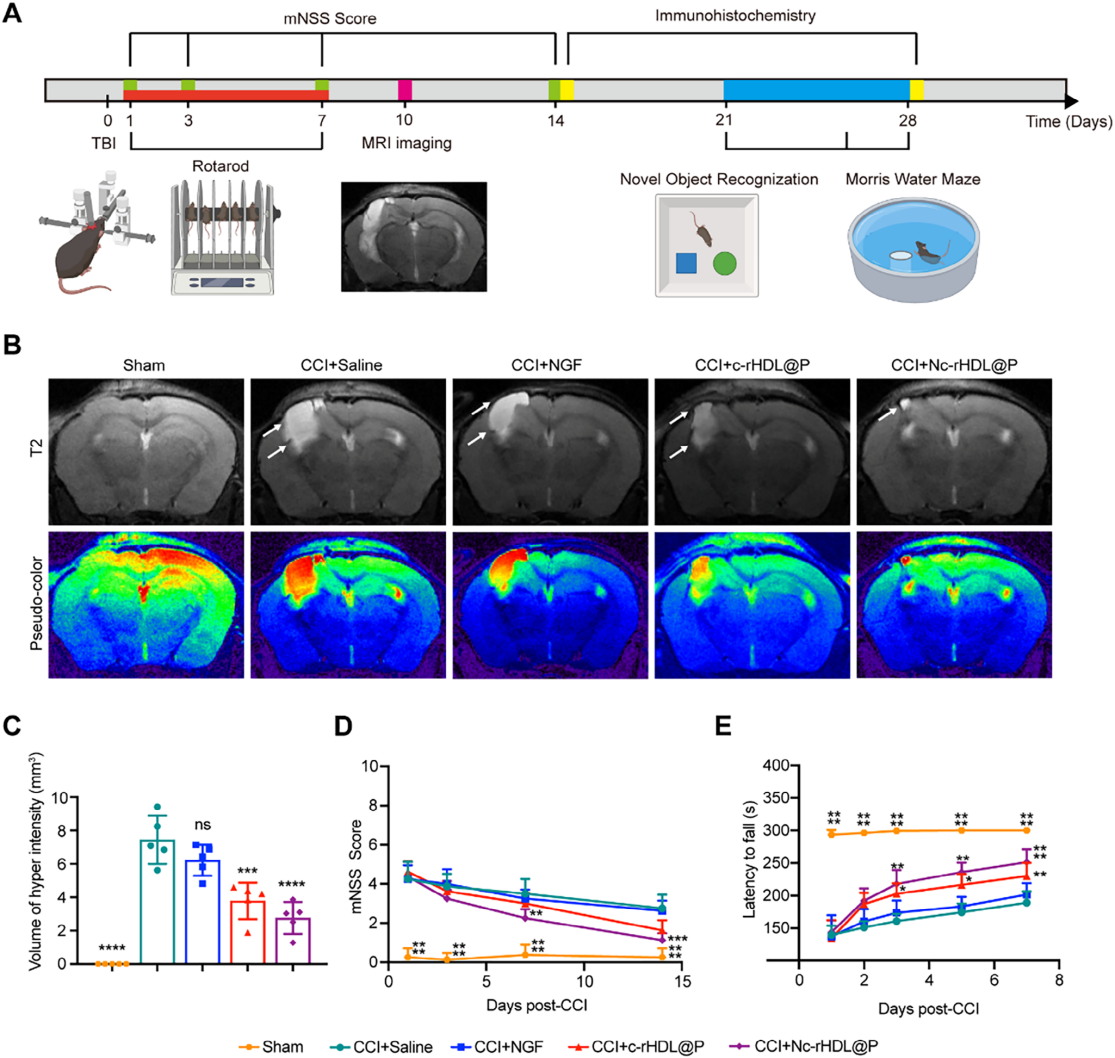
Nc‐rHDL@P improved sensorimotor function and rescued cognitive deficits in the CCI model mice. A) Schedule of CCI, treatment, and therapeutic evaluation. The schematic diagram was created in BioRender. Weng, W. (2025) (https://BioRender.com/25xnrv1). B) T2 MRI images of CCI model mice after the treatment of free NGF or NGF formulations. C) Semi‐quantitative analysis of the volume of hyperintensity. D) Neurological impairments measured by mNSS score after CCI. E) The latency to fall in the rotarod test after CCI. Data represent mean ± SD, *n* ≥ 3. ^*^ *p* < 0.05, ^**^ *p* < 0.01, ^***^ *p *< 0.001, and ^****^ *p* < 0.0001. ns, not significant. One‐way ANOVA with Bonferroni's multiple comparisons test was used for (C). Two‐way ANOVA with Bonferroni's multiple comparisons test was used for (D) and (E).

Having substantiated the ameliorative effects of Nc‐rHDL@P on inflammatory microenvironment and neuropathological damage in the CCI model mice, we proceeded to interrogate its therapeutic potential in rescuing sensorimotor dysfunction and cognitive deficits. After treatments with different NGF formulations, sensorimotor function was measured via the modified neurological severity score (mNSS) along with the rotarod test, and the cognitive function was measured with the novel object recognition (NOR) test followed by the Morris water maze (MWM) test (**Figure** **5**A). Given the association between sensorimotor deficits and cognitive impairments of CCI mice with the contusion of the primary motor cortex, association cortex, and the septal pole of the hippocampus, structural magnetic resonance imaging (MRI) with T2‐weighted imaging (T2WI) was conducted 10 days after CCI to assess the lesion‐associated hyperintensity signals (Figure 5B,C). Treatment with Nc‐rHDL@P reduced the volume of T2 hyperintensity from 7.44 to 2.76 mm^3^ in the ipsilateral region (Figure 5C). In contrast, treatment with free NGF (6.22 mm^3^) resulted in minimal reduction in the volume of T2 hyperintensity, showing no significant difference when compared to mice treated with saline (7.44 mm^3^). The volume of T2 hyperintensity in the c‐rHDL@P‐treated group was 3.78 mm^3^. Although this reduction was not as pronounced as that observed in the Nc‐rHDL@P‐treated group, it still indicated that the empty vector possessed certain therapeutic effects and could exert synergistic therapeutic effects with loaded NGF.

After demonstrating the restorative effects of Nc‐rHDL@P on damaged brain regions, we assessed the animals’ sensorimotor function using the mNSS, a task‐based examination for detecting sensorimotor impairments, and the rotarod test, a widely used paradigm for assessing balance and motor coordination.^[^ ^53^, ^54^, ^55^ ^]^ After treatment, free NGF caused no significant change in both mNSS score and rotarod test, while the c‐rHDL@P treatment group showed 21.61% longer latency to fall off, with no significant change in mNSS score (Figure 5D,E). Compared with the saline‐treated CCI mice, the mice treated with Nc‐rHDL@P exhibited significantly lower mNSS scores, decreasing from 4.38 to 1.13, indicating improvement in sensorimotor function (Figure 5D). This substantial reduction in mNSS scores was indicative of a significant recovery in the sensory and motor functions of the treated mice. Following the mNSS findings, the rotarod test revealed that Nc‐rHDL@P‐treated CCI mice had superior motor function as quantified with 32.91% longer latency to fall off the rotarod (Figure 5E). This enhanced performance in the rotarod test underscored the efficacy of Nc‐rHDL@P in promoting motor function recovery of CCI mice.

**Figure 6:**
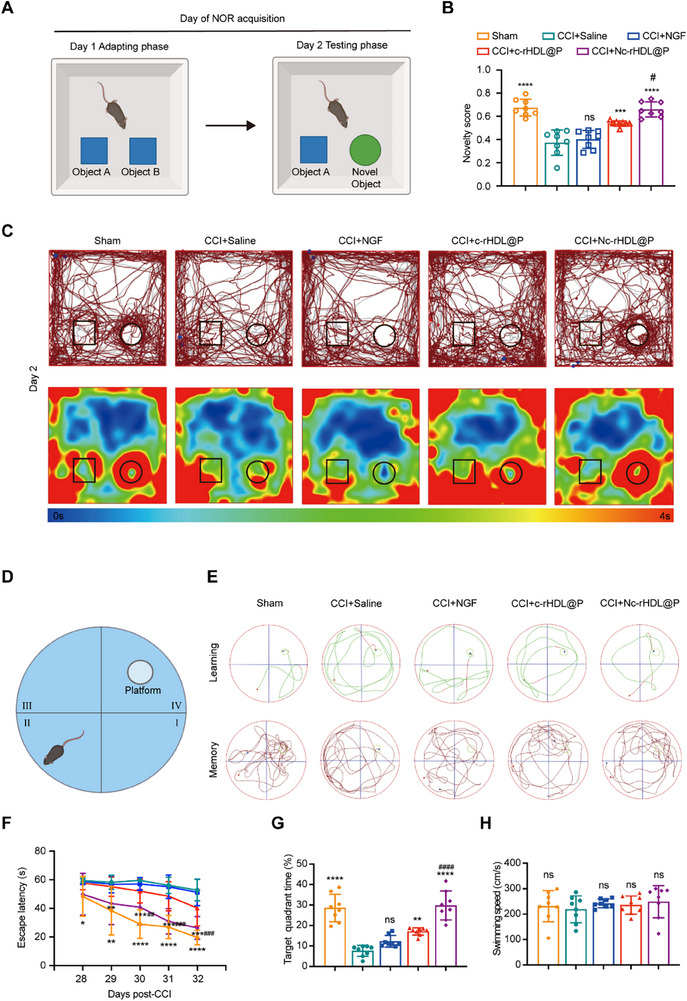
Nc‐rHDL@P rescued cognitive deficits in the CCI model mice. A) The schematic illustration of the NOR test. B) The novelty score of CCI model mice after treatment of free NGF or NGF formulations. C) The trajectory in the testing phase. D) The schematic illustration of the MWM. E) The path map for the learning and memory phase of the CCI model mice after treatment of free NGF or NGF formulations. F) The latency in the hidden platform test. G) The time spent on exploring the target quadrant. H) The swimming speed in the probe trial. Data represent mean ± SD, *n* ≥ 3. ^*^ *p* < 0.05, ^**^ *p* < 0.01, ^***^ *p* < 0.001, and ^****^ *p* < 0.0001 indicated that there was a significant difference compared to the CCI+saline group. ^#^ *p* < 0.05, ^##^ *p* < 0.01, ^###^ *p* < 0.001, and ^####^ *p* < 0.0001 indicated that there was a significant difference between the CCI+c‐rHDL@P and CCI+Nc‐rHDL@P groups. ns, not significant. One‐way ANOVA with Bonferroni's multiple comparisons test was used for (B), (G), and (H). Two‐way ANOVA with Bonferroni's multiple comparisons test was used for (F). (A) and (D) were created in BioRender. Weng, W. (2025) (https://BioRender.com/25xnrv1).

To investigate the recovery of the hippocampal lesion, we quantified the cognitive function of the post‐contusion animals with NOR and MWM tests. The NOR test exploits the innate exploratory drive of rodents to quantify hippocampal‐dependent recognition memory through a quantitative ethological metric comparing temporal investment in novel versus familiar objects (**Figure** **6**A).^[^ ^56^ ^]^ CCI mice treated with c‐rHDL@P and Nc‐rHDL@P exhibited 44.27% and 76.94% higher novelty scores, respectively, than the saline‐treated group, demonstrating better recognition memory to explore the new objects, which was supported by the trajectory of movements (Figure 6B,C). In contrast, free NGF‐treated CCI mice showed no significant improvement in novelty scores (Figure 6B). Additionally, the MWM test assessed the spatial learning and memory capabilities of the CCI model mice (Figure 6D).^[^ ^57^ ^]^ The results revealed that Nc‐rHDL@P‐treated mice spent 293.72% more time in the target quadrant compared to the control group, indicating a substantial enhancement in spatial memory retention (Figure 6E–G). This level of improvement far surpassed that of the c‐rHDL@P‐treated group, which showed a 128.74% increase in target quadrant time (Figure 6G). In contrast, free NGF treatment failed to induce any significant amelioration in spatial learning and memory, as evidenced by the trajectory patterns, escape latency during the learning phase, and the time spent in the target quadrant (Figure 6E–G). Moreover, there was no statistical difference in swimming speed among all groups during the probe trial, demonstrating that the observed disparities were not caused by differences in motor functions (Figure 6H). The findings demonstrated that Nc‐rHDL@P significantly enhanced the cognitive function of CCI mice. To sum up, the findings revealed that Nc‐rHDL@P outperformed the empty carrier in enhancing both cognitive and sensorimotor functions. It demonstrated great potential as a therapeutic strategy for addressing the multifaceted deficits associated with CCI injury.

To thoroughly evaluate the safety profile of Nc‐rHDL@P, we undertook a comprehensive series of assessments. These included histopathological examination of major organs using hematoxylin and eosin (H&E) staining, as well as detailed analysis of serum biochemical markers. H&E staining results showed no pathological changes in the major organ sections excised from mice treated with various formulations (Figure S18A, Supporting Information). This finding indicated the excellent biosafety of Nc‐rHDL@P, as it did not induce any significant tissue damage or inflammation in the major organs. Also, the negligible changes in alanine transaminases (ALT), aspartate transaminases (AST), creatine kinase (CK), lactate dehydrogenase (LDH), blood urea nitrogen (BUN), and creatinine (CREA) suggested little renal and hepatic damage after Nc‐rHDL@P treatment, further supporting its safety profile (Figure S18B, Supporting Information). Collectively, these results indicated that Nc‐rHDL@P can safely alleviate the secondary brain damage of TBI, thereby mitigating sensorimotor and cognitive dysfunction.

## 3 Conclusion

In summary, we have developed a neurophilic biomimetic lipoprotein that integrates NGF protection through a cell matrix‐like core, BBB penetration via an rHDL shell, and neuron targeting using an αRDP peptide, thereby achieving efficient packaging of NGF. This lipoprotein effectively safeguarded NGF's bioactivity, successfully traversed the BBB, and released NGF specifically in TBI‐affected brain regions. Consequently, the resultant nanocomplex robustly promoted neuronal differentiation and neurite outgrowth, reduced neuronal damage, enhanced neurogenesis, attenuated neuroinflammation and glial scar formation, and ultimately improved motor function, spatial learning, and memory recovery in TBI mice.

Overall, this versatile neurophilic biomimetic lipoprotein represents a significant advancement in neurotrophin delivery, with potential applications extending beyond NGF to other therapeutic protein cargos for TBI and various neurological disorders. While the present study focused on Nc‐rHDL@P's efficacy in TBI, future research could investigate its applicability to other neurological conditions—such as stroke and neurodegenerative diseases—to further validate its potential for broad‐spectrum neuroprotection and neural repair. Further optimization of the lipoprotein design, development of more specific targeting ligands (e.g., αRDP peptide‐based systems) to enhance selectivity for damaged cells, and investigation of combination therapies with neuroprotective agents (e.g., antioxidants, anti‐inflammatories) or physical therapies could refine its therapeutic scope. These efforts may ultimately establish this platform as a versatile strategy for delivering a broad range of protein‐based neurotherapeutics, thereby significantly enhancing its clinical potential.

## 4 Experimental Section

### Cell Culture

bEnd.3 (Cat No.FH0356, RRID: CVCL_0170), HT22 (Cat No.FH1027, RRID: CVCL_0321), RSC96 (Cat No.FH1100, RRID: CVCL_4694), and PC12 (Cat No.FH0413, RRID: CVCL_0481) cell lines were obtained in March 2024 from the Cell Institute of Chinese Academy of Sciences (Shanghai, China). bEnd.3 and HT22 cells were cultured in Dulbecco's Modified Eagle's Medium (DMEM) (Gibco, Grand Island, NY, USA) supplemented with 10% fetal bovine serum (FBS) (Gibco, Grand Island, NY, USA) and 1% penicillin/streptomycin at 37 °C in a humidified atmosphere containing 5% CO_2_. PC12 cells were cultured in RPMI 1640 (Hyclone, Logan, Utah, USA) and supplemented with 10% horse serum, 5% FBS, and 1% penicillin/streptomycin for proliferation. PC12 cells were cultured in RPMI 1640 and supplemented with 1% horse serum and 1% penicillin/streptomycin but without FBS for differentiation. The hNSCs were procured in May 2024 from Shanghai Angecon Biotechnology Co., Ltd, which were isolated from embryos and then induced. Upon receipt, the hNSCs suspension was centrifuged at 300 g for 5 min. The supernatant was discarded, and the cells were resuspended and seeded into T25 low‐attached flasks with 4 mL expansion medium. The expansion medium contained DMEM/F12 (Gibco, Grand Island, NY, USA), 2% B27 supplement (Gibco, Grand Island, NY, USA), 20 ng mL^−1^ of basic fibroblast growth factor (bFGF) (Gibco, Grand Island, NY, USA), 20 ng mL^−1^ of epidermal growth factor (EGF) (Gibco, Grand Island, NY, USA), and 1% penicillin–streptomycin to promote growth. The hNSCs grew as neurospheres, and one flask was supplemented with 1 mL expansion medium every two days during hNSCs expansion. The differentiation medium for hNSC contained DMEM/F12, 2% B27 supplement, and 1% penicillin–streptomycin. RSC96 were cultured in DMEM supplemented with 10% FBS and 1% penicillin/streptomycin at 37 °C in a humidified atmosphere containing 5% CO_2_. All cell lines above underwent species authentication and mycoplasma testing before the experiments.

### Animals and CCI Mouse Model

C57BL/6 mice (RRID: MGI:2159769) were sourced from Shanghai SLAC Laboratory Animal Co., Ltd. (Shanghai, China). Thy1‐GFP transgenic mice were generously provided by Professor Xiao‐Ling Gao from the School of Medicine, Shanghai Jiao Tong University. The animals were housed in a specific pathogen‐free (SPF) facility under a 12 h light/12 h dark cycle, with an ambient temperature of 20–25 °C and humidity of 40%–70%. All animal experiments were conducted with the approval of the Institutional Animal Care and Use Committee of Renji Hospital, School of Medicine, Shanghai Jiao Tong University (Approval number: 2023–106).

Male C57BL/6 mice (8 weeks old) were used to establish the CCI model. Anesthesia was induced with 3% isoflurane delivered in a nitrous oxide: oxygen carrier gas mixture (7:3 volume ratio) and maintained via a nose cone system with 1.5% isoflurane. Following adequate anesthesia, animals were secured in a stereotaxic apparatus (R.W.D. Co., Ltd., Shenzhen, China). A 4 mm‐diameter craniotomy was performed using a high‐speed drill over the left parietal bone, midway between the temporalis muscle and the central sagittal suture, within the bregma‐lambda interaural plane between bregma and lambda. The bone flap was carefully removed to expose the underlying dura mater. The impactor piston was positioned at a 15°–20° angle relative to the sagittal plane, ensuring the impactor tip remained perpendicular to the cortical surface. Controlled cortical injury was induced using a PinPoint PCI3000 Precision Cortical Impactor (Hatteras Instruments, Cary, NC, USA) equipped with a 2.5 mm‐diameter electromagnetic impactor tip. Impact parameters were set as follows: deformation depth 1 mm, velocity 3 m s^−1^, and dwell time 100 ms. After the contusion, the surgical site was sutured in layers, and animals were allowed postoperative recovery on a 37 °C heating pad to maintain core body temperature at 36–37 °C. Sham‐operated animals underwent identical surgical procedures, excluding the impact protocol.

### Preparation and Characterization of the Nanoparticles

DMPC and DOPA were purchased from Avanti Polar Lipids (Alabaster, AL, USA). It should be noted that DOPA is an unsaturated phospholipid, which is highly susceptible to oxidation. Therefore, its stock solution is prepared at a higher concentration of 20 mm in chloroform to maintain stability. First, DMPC (6 µmoL) and DOPA (4 µmoL) were mixed and dissolved in 4 mL chloroform before being transferred to a rotary evaporator. The chloroform solvent was evaporated, and a thin film was formed at room temperature under the Büchi rotavapor R‐200 (Büchi, Germany) for 1 h. The lipid film was rehydrated at 40 °C by adding 4 mL of tri‐distilled water and vortexing intermittently for 10 min. The resulting liposomes (Lipo) were probe‐sonicated (Scientz Biotechnology Co. Ltd., China) at 100 W output for 10 min to improve particle size uniformity. Second, NGF (Sino Biological, Shanghai, China) was mixed with HA and PRTM (molar ratio of 112.2:1:30.6) at room temperature for 10 min to form the Nc. Third, Nc was incubated with the preformulated Lipo for 30 min to form a lipid‐encapsulated core‐shell complex. Then the above complex was incubated with ApoE3 (14.5 nmoL, Pepro Tech, Rocky Hill, NJ, USA) and αRDP (AC‐FAEKFKEAVKDYFAKFWD‐PEG12‐GNSARKGRSNTFIDCPTGPRPNEPMWITY, GL Biochem Ltd., 6.49 nmoL) sequentially at 37 °C and 120 rpm for 48 h to obtain Nc‐rHDL@P. RBITC‐, DiI‐ or DiR‐labeled Lipo was also prepared using the same procedure. Specifically, RBITC, DiI or DiR was added to the chloroform solution containing DMPC and DOPA during the preparation process. NGF was labeled with Alexa Fluor 647 or FITC following the guidelines provided in the protein labeling kit (Thermo Fisher Scientific, USA). The Alexa Fluor 647‐ or FITC‐labeled NGF was subsequently purified through a purification column (Cytiva, USA) and then stored at −80 °C until further experiments were conducted.

To verify the successful assembly of Nc‐rHDL@P, fluorescence resonance energy transfer (FRET) analysis was conducted. In this experiment, RBITC‐labeled Lipo was employed to quench the fluorescence of FITC‐labeled NGF. The fluorescence spectra were recorded using a microplate reader (Thermo Multiskan MK3, USA), with an excitation wavelength set at 470 nm and an emission wavelength range of 500–700 nm. The morphology and size of Nc‐rHDL@P and its related formulations were examined using H‐7650 transmission electron microscopy (Hitachi, Inc., Japan) and cryo‐transmission electron microscopy (Tecnai G2 F20, FEI, USA). For DeltaVision OMX SR imaging, a drop of antifade mounting medium (P0126, Beyotime, China) was first added to a glass‐bottomed dish, followed by the addition of Nc‐rHDL@P. Subsequently, colocalization analysis was performed using the DeltaVision OMX SR system. For AFM imaging, the prepared samples were carefully deposited onto a freshly cleaved mica sheet to ensure a uniform and stable substrate. Following deposition, the samples were gently dried using a stream of nitrogen gas to remove any residual moisture and maintain the integrity of the sample structure. The particle size distribution, zeta potential, and concentration of Nc‐rHDL@P and its related formulations were assessed using the Zetasizer Nano‐ZS90 and Nanosight NS300, both from Malvern Instruments (U.K.). The stability of Nc‐rHDL@P was evaluated by monitoring changes in particle size distribution, zeta potential, and PDI. These measurements were taken once every other day, for a total of five assessments. To evaluate the encapsulation efficiency of NGF in c‐rHDL@P, Alexa Fluor 647‐labeled NGF was used as a marker. For quantitative analysis, Alexa Fluor 647‐labeled Nc‐rHDL@P was dissolved in an equal volume of anhydrous ethanol at 65 °C for 10 min to release the encapsulated protein cargo. The resulting solution was then analyzed using a microplate reader (Thermo Multiskan MK3, USA).

To analyze the release kinetics of Nc‐rHDL@P., native NGF and Nc‐rHDL@P at a final concentration of 1000 ng mL^−1^ were incubated at different pH conditions (pH 6.0 and pH 7.4) to simulate the injured condition at 37 °C for 24 h. Additionally, cerebrospinal fluid (CSF) from TBI and non‐TBI patients was collected. Native NGF and Nc‐rHDL@P at a final concentration of 1000 ng mL^−1^ were incubated in CSF. The mixture in each group was sampled at 0, 4, 8, 12, 16, and 24 h. The samples were measured using an ELISA kit with a standard curve established per manufacturer's instructions. The clinical protocol for CSF collection in TBI patients received ethical approval from the Institutional Review Board of Renji Hospital, Shanghai Jiao Tong University School of Medicine (Approval No: LY2023‐018‐B) and was prospectively registered at ClinicalTrials.gov (Identifier: NCT05778123). Three TBI patients and three age‐matched controls with hydrocephalus at Renji Hospital were recruited between June 2023 and March 2024. Samples of TBI patients were collected within 6 h post‐injury during emergency neurosurgical procedures. All specimens were anonymized immediately after acquisition.

### CCK‐8)Assay

Cell viability was measured by CCK‐8 (Vazyme, Nanjing, China). PC12 cells were seeded on 96‐well plates and treated with H_2_O_2_ (50 µm) for 2 h. HT‐22 cells were seeded on 96‐well plates and treated with H_2_O_2_ (200 µm) for 2 h. After incubation of NGF, Nc‐Lipo, Nc‐rHDL, and Nc‐rHDL@P for 12 h, 10 µL CCK‐8 reagent was added to each well, and the plate was incubated at 37 °C for 2 h. Absorbance was measured at 450 nm using a microplate reader (Molecular Devices, Sunnyvale, CA, USA).

### Differentiation Assay

PC12 cells were cultured in RPMI 1640 and supplemented with 10% horse serum and 5% FBS for proliferation. Before the treatment, PC12 cells were switched to differentiation media consisting of RPMI 1640, 1% horse serum, and 1% penicillin/streptomycin for 12 h. Then, native NGF, and various NGF formulations were added with a final concentration of 100 ng mL^−1^. PC12 cells were imaged at three random regions of each well under phase contrast for 7 days. The number of PC12 cells with extended neurites (> 50 µm) was then counted from the images taken at different regions.

For the process of hNSCs neuronal induction, the neurospheres were dissociated into a single‐cell suspension and plated on coverslips that had been previously coated with 0.05 mg mL^−1^ poly‐L‐lysine at a density of 2 × 10^4^ cells cm^−2^. Subsequently, the cells were cultured in an expansion medium for 48 h, to allow for adequate recovery from passage. Once the recovery period had elapsed, the cultured medium was changed to a neural differentiation medium, native NGF and various NGF formulations were added with a final concentration of 100 ng mL^−1^. Following 7 days of culture, the cells were collected for immunofluorescence.

On day 7, the cells were washed, fixed with paraformaldehyde (4%, w/v), permeated with Triton X–100 (0.5%, w/v), blocked with BSA (5%, w/v), and stained with MAP2 (Cell Signaling Technology, USA) for neural dendrites and DAPI for nuclei. Cells were then imaged under confocal microscopy (TCS SP8, Leica Camera, AG, Germany). Quantitative cellular analysis was performed with a KineticScan HCS reader (Thermo Fisher Scientific, Waltham, MA, USA).

RSC96 cells were passaged for expansion upon reaching 80% confluency. The native NGF and various NGF formulations were supplemented with 100 ng mL^−1^. NGF and various NGF formulations were added after half the medium replacement every 2 days. For morphological observation of cell adhesion at day 3, cells were fixed, permeabilized, stained with Rhodamine Phalloidin (Abcam, Cambridge, UK) and DAPI, and subjected to imaging under a fluorescence microscope. Quantification of dendrite length was performed with ImageJ.

### mRNA Isolation and RT‐qPCR

Total RNA was extracted from RSC96 cells after 3‐day treatment using TRIzol (Vazyme, Nanjing, China). RNA purity and concentration were determined using a Nanodrop spectrophotometer (Nanodrop Technologies, Wilmington, DE). One microgram of RNA was reverse transcribed to synthesize cDNA using Hifair II 1st Strand cDNA Synthesis Kit (Yeasen, Shanghai, China). To measure cytokine expression, quantitative PCR was performed using the AceQ qPCR SYBR Green Master Mix (Vazyme, Nanjing, China). Target cDNAs and reference cDNAs (GAPDH) were amplified by PCR using TaqMan gene expression assays for sequence‐specific primers purchased from Sangon (Sangon Biotech, Shanghai, China). The expression levels of NGF were focused on (forward primer: 5′‐TGATCGGCGTACAGGCAGA‐3′; reverse primer: 5′‐GAGGGCTGTGTCAAGGGAAT‐3′), GDNF (forward primer: 5′‐CCGGACGGGACTCTAAGATGA‐3′; reverse primer: 5′‐GTCAGGATAATCTTCGGGCATATT‐3′), CNTF (forward primer: TTTCGCAGAGCAAACACCTCT; reverse primer: TGCTAGCCAGATAGAACGGCTAC) mRNA as markers. Quantitative real‐time PCR was performed with a 384‐well optic plate on a Lightcycler 480 instrument (Roche Diagnostics, Basel, Switzerland). Data were analyzed using the comparative threshold cycle (Ct) method, and results are expressed as relative fold change to control target mRNA expression. The primer sequences of GAPDH were 5′‐ATGGTGAAGGTCGGTGTG‐3′ (forward) and 5′‐TGTAGTTGAGGTCAATGAAGGG‐3′ (reverse).

### Quantification of Cellular Uptake of Alexa Fluor 647‐Labeled Nc‐rHDL@P by bEnd.3 Cells

bEnd.3 cells were plated at a density of 0.5 × 10⁴ cells per well in a glass‐bottom 96‐well plate using DMEM supplemented with 10% FBS. Once the confluence reached ≈75%, the cells were treated with Alexa Fluor 647‐labeled Nc‐rHDL@P (containing 20 µg mL^−1^ NGF) in DMEM for 4 h at 37 °C. Following incubation, the bEnd.3 cells were washed three times with PBS and then fixed with 4% PFA for 15 min at room temperature. The nuclei were subsequently stained with DAPI. The internalization of Alexa Fluor 647‐labeled Nc‐rHDL@P by bEnd.3 cells was quantified using ImageJ software.

### Transwell Migration Assay

In the development of the in vitro BBB model, a monolayer of bEnd.3 cells were meticulously cultured on transwell membranes made of permeable polyester within a 24‐well plate (Corning, NY, USA). These cells were maintained in DMEM medium supplemented with 10% FBS for 7 days to ensure proper growth and development. The integrity of the cell monolayer was assessed using TEER measurements and further validated through immunofluorescence staining. Monolayers with TEER values greater than 200 Ω·cm^2^ were selected for subsequent experiments. To visualize the cellular structure and distribution, the monolayers were fixed, permeabilized, stained with Rhodamine Phalloidin (Abcam, Cambridge, UK) and DAPI, and imaged using a fluorescence microscope.

For the permeability studies, Alexa Fluor 647‐labeled Nc‐rHDL@P, Nc‐rHDL, Nc‐Lipo, and free NGF were introduced into the upper chamber of the transwell system. The filtrate in the lower chamber was collected at specific time points (1, 3, 6, and 12 h), and the fluorescence intensity of the filtrate was measured using a microplate reader (Thermo Multiskan MK3, USA) to evaluate the transport efficiency of these substances across the BBB model. Based on normalizing the fraction of NGF formulations that penetrated blank filters, the percentage of formulations that penetrated the cell‐coated monolayer were calculated.

### In Vivo Biodistribution of Nc‐rHDL@P in Mice

To study the biodistribution of Nc‐rHDL@P, the CCI mice were randomly separated into four groups. The in vivo accumulation of Nc‐rHDL@P in the CCI mice was visualized using DiR‐labeled lipid shell and AF647‐labeled NGF following intravenous administration at the NGF dose of 2.5 mg kg^−1^ via an in vivo imaging system. The groups were as follows: 1) the CCI + saline group, 2) the CCI + Native NGF group, 3) the CCI + Nc‐Lipo group, 4) the CCI + Nc‐rHDL group, 5) the CCI + Nc‐rHDL@P group. At 4 h post‐injection, the mice were sacrificed, with the peripheral organs and the brain tissue collected. After being rinsed with saline, the organs were studied via an in vivo imaging system (IVIS Spectrum, PerkinElmer, USA).

For brain section analysis, the Thy1‐GFP mice, which had green fluorescent protein‐labeled neurons, were chosen for studying the cellular uptake of Nc‐rHDL@P and the ability of neural targeting. At 4 h post‐injection, the mice were anesthetized, and the heart was perfused with saline and 4% paraformaldehyde. Then the entire brains were collected, fixed in 4% paraformaldehyde, dehydrated in 10% and 30% sucrose solution, embedded in OCT (Sakura, Torrance, CA, USA), frozen at −25 °C, and sectioned at 20 µm before observation under a confocal microscope. Three mice and at least 3 brain tissue pieces were included in each group. Fluorescence images were captured by a Leica microscope with consistent imaging settings.

The pharmacokinetic data of NGF were assessed by monitoring the residual NGF level in the brain tissue after intravenous administration. Briefly, C57BL/6 mice were injected with free NGF or various NGF formulations via the tail vein at a dose of 2.5 mg kg^−1^ of body weight after CCI for 30 min. The brain tissue samples from CCI mice injected with saline without the injection of NGF formulations were sampled as the negative control. All the mice were transcardially perfused with PBS before the injured brain tissue was collected. The brain tissues were snap‐frozen in liquid nitrogen and mechanically homogenized using a cooled mortar and pestle. The powder was transferred to 5 mL polypropylene centrifuge tubes and subjected to lysis with four tissue volume equivalents of ice‐cold buffer containing 8 m urea and 1% protease inhibitor cocktail. Cellular disruption was enhanced through three intermittent cycles of high‐intensity ultrasonication (10 s pulses with 30 s cooling intervals) performed on ice using a probe sonicator (Scientz, China). Lysates were purified by sequential centrifugation at 12000 × g for 10 min at 4 °C. The protein‐containing supernatant was collected, with quantification performed using a bicinchoninic acid (BCA) assay kit (E112‐01, Vazyme, China) following standardized protocols. The residual NGF level in the brain tissue samples was then determined by ELISA (RayBiotech, USA) with a standard curve established according to the manufacturer's instructions.

### Drug Treatment of the CCI Model Mice

CCI mice were intravenously administered with saline, NGF (1 mg kg^−1^), c‐rHDL@P (at the same concentration as Nc‐rHDL@P), or Nc‐rHDL@P (1 mg kg^−1^) once every 3 days, for a total of ten doses (*n* = 8 per group). As controls, both CCI model mice and sham‐operated mice received saline (*n* = 8 per group).

### Inflammatory Cytokine Expression Levels

In accordance with the manufacturer's protocol, brain homogenate was collected for ELISA analysis to assess the impact of various treatments on the expression of IL‐6, IL‐1β, and TNF‐α 72 h post‐CCI. Specifically, the supernatant of the brain lysate was clarified by centrifugation at 10 000 g for 1 min. Subsequently, the protein concentration was normalized using a BCA assay kit. The samples were then loaded into 96‐well plates pre‐coated with an inflammatory cytokine‐binding protein and incubated at 37 °C for 30 min. The plates were incubated with a primary antibody after thorough washing, followed by a horseradish peroxidase (HRP)‐conjugated secondary antibody. Finally, the HRP substrate was added, and the absorbance was measured at 490 nm to determine the levels of IL‐6, IL‐1β, and TNF‐α.

### mNSS Assessment

Motor functional recovery was evaluated using the mNSS, a validated 10‐task behavioral battery assessing integrated sensorimotor performance across three domains: limb motility, reflex integrity, and postural stability. Serial assessments were conducted on postoperative days 1, 3, 7, and 14 employed a deficit‐weighted scoring system where task failure accrued one point per item (maximum score = 10), with neurological impairment severity categorized as mild (1‐4 points), moderate (5 points), or severe (6–8 points) based on established thresholds for CCI models. To ensure objectivity, two independent investigators blinded to experimental groupings performed all evaluations.

### Rotarod Test

Motor function assessment was conducted using a Rotor‐Rod locomotor testing system (San Diego Instruments, Inc., San Diego, CA, USA). The training phase involved three consecutive days of habituation under standardized acceleration parameters (0–40 rpm over 150 s followed by a 150 s sustained velocity phase at 40 rpm) for both CCI and sham groups. Postoperative evaluations commenced at postoperative day 1 and continued at day 3, day 5, and day 7, with daily triplicate trials spaced by ≥30 min inter‐trial intervals to minimize fatigue‐related confounds. Behavioral endpoints were defined as trial termination upon complete dismount or passive rotation (>2 full revolutions while maintaining grip), while successful task completion required sustained ambulation throughout the 300 s trial duration. Performance metrics were quantified as mean fall latency (seconds) across three consecutive trials per testing day.

### MRI

High‐resolution neuroimaging was performed using a 7.0 Tesla Bruker BioSpec 70/20 USR preclinical MRI system (Bruker Biospin GmbH, Ettlingen, Germany) with an integrated physiological monitoring system. An orthogonal coil (inner diameter 72 mm) was used to generate radio‐frequency pulses, while a four‐channel phased‐array surface receiver coil was employed for signal detection. Animal physiological stability was maintained through a closed‐loop water‐circulating heating pad, with continuous monitoring of respiratory rate and rectal temperature via an MR‐compatible monitoring system (Model 1025, SA Instruments, Inc.). T2‐weighted anatomical imaging was acquired using a turbo rapid acquisition with relaxation enhancement (RARE) sequence with the following parameters: repetition time (TR) = 3000 ms, echo time (TE) = 46.67 ms, field of view (FOV) = 20 × 20 mm^2^, matrix size = 256 × 256, slice thickness = 0.6 mm (30 contiguous slices), number of averages (NEX) = 4. Raw DICOM datasets were reconstructed using the manufacturer's proprietary software suite (ParaVision 6.0.1, Bruker Biospin) and subsequently analyzed for apparent diffusion coefficient (ADC) mapping through a standardized pipeline integrating ImageJ with the MRI Analysis Calculator plugin (v2.1.0).

### NORt)

A NOR task was implemented to assess spatial learning and memory consolidation, preceding MWM evaluation. During two habituation trials, mice were placed in an area containing two identical objects (same in shape, color, and smell) and allowed to explore freely for 300 s. The test trial introduced a novel object (different in shape, color, and smell) positioned equidistantly from the subject. Each 300 s trial quantified exploration time, with novelty score calculated as: Novel object exploration duration / (Novel + Familiar object exploration duration).

### MWM Test

All mice underwent the MWM test following CCI. The spatial memory assessment was performed in a 1.2 m diameter circular black polypropylene tank filled with opaquely tinted water, where four unique geometric patterns functioned as stable extra‐maze visual cues fixed to the tank walls in cardinal orientations. A 10 cm transparent platform, submerged 2 cm beneath the water surface, was permanently stationed in the predefined target quadrant. Over five consecutive training days, animals underwent daily training sessions to learn platform location through trials initiated from randomized entry positions along the tank edge. On postoperative day 6, a probe trial involving platform removal was conducted for 60 s, during which swim paths were recorded using a video tracking system. Analyzed metrics included escape latency (time to reach the platform area), path efficiency (ratio of optimal to actual swimming distance), number of platform crossings, and time spent in the target quadrant. Before the formal testing, a 120 s screening session without the platform was conducted to ensure comparable swimming speed.

### Immunofluorescence and Immunohistochemical Analysis

At the end of the experiment, all experimental mice were euthanized, followed by transcardial perfusion. Brain tissues were harvested and sequentially processed through standardized neurohistological protocols: 24 h fixation in 10% neutral‐buffered formalin at 4 °C, dehydration in 30% sucrose solution, paraffin embedding, and coronal sectioning at 4 µm thickness. Antigen retrieval was achieved through citrate buffer (pH 6.0) incubation at 95 °C for 15 min, followed by endogenous peroxidase quenching with 0.3% hydrogen peroxide/methanol. Sections were blocked with 5% normal goat serum containing 0.1% Triton X‐100 in PBS for 1 h at room temperature. Primary antibodies against NeuN, IBA1, and GFAP (Cell Signaling Technology, USA) were incubated overnight at 4 °C in blocking solution. For immunofluorescence analysis, the antigens were detected using fluorescent‐labeled secondary antibodies. For immunohistochemical analysis, antigen detection was performed using secondary antibodies and the standard ABC‐DAB method. The sections were then subjected to quantitative analysis using ImageJ software, which facilitated a detailed assessment of the distribution and activation states of neurons, microglia, and astrocytes.

### Nissl Staining

After treatment with different formulations, the brains from both CCI model mice and sham‐operated mice were prepared for Nissl staining. Paraffin‐embedded sections were dewaxed in water. The sections were then stained in toluidine blue solution for 1–2 min, followed by washing with water. They were subsequently differentiated using 0.5% glacial acetic acid and dried in an oven at 65 °C for over 4 h. The sections were then immersed in clean xylene I for ≈2 min, followed by clean xylene II for 15 min, and finally sealed with a neutral resin.

### Golgi‐Cox Staining

Following treatment with various formulations, the brains from both CCI model mice and sham‐operated mice were prepared for Golgi‐Cox staining. This procedure was carried out using the FD Rapid Golgi Stain Kit (Catalog No. PK‐401) from FD NeuroTechnologies (USA), strictly adhering to the manufacturer's instructions. After staining, the resulting slides were examined under a Leica DM3000 microscope to observe the detailed morphology of the neurons.

### Biosafety Evaluation

To evaluate the safety of Nc‐rHDL@P during treatment, CCI mice were intravenously injected with the corresponding NGF formulations or free NGF. Mice that received saline served as controls. After euthanasia, major peripheral organs (heart, liver, spleen, lung, and kidney) were harvested, sectioned, and stained with hematoxylin and eosin (H&E). Serum samples were carefully extracted from the mice following the intravenous injection of the NGF formulations. These samples were then subjected to a series of biochemical analyses to assess the potential impact of the treatments on vital organ functions. Specifically, liver function was evaluated by measuring the levels of AST, ALT, CK, and LDH. Simultaneously, renal function was assessed by determining the BUN and CREA.

### Statistical Analysis

All data are shown as the mean ± standard deviation (SD) unless otherwise specified. All statistical analysis was performed with Prism 9 (GraphPad Software). For comparisons between two groups, the unpaired Student's *t*‐test (two‐tailed) was used. For analyses involving multiple groups, one‐way ANOVA or two‐way ANOVA followed by Bonferroni testing was applied. The significance level was chosen at *p* < 0.05.

## Conflict of Interest

The authors declare no conflict of interest.

## Supporting information



Supporting Information

## Data Availability

The data that support the findings of this study are available from the corresponding author upon reasonable request.
