# Supplementary material for: Neurophilic Biomimetic Lipoprotein‐Mediated Targeted Nerve Growth Factor Delivery for Traumatic Brain Injury Therapy
Source: Adv Sci (Weinh). 2025 Aug 18;12(42):e09405. doi: 10.1002/advs.202509405 (PMC12622449; doi:10.1002/advs.202509405)
Supplement: Supplementary file 1 — Supporting Information [file ADVS-12-e09405-s001.docx]

**Supporting Information**

Neurophilic Biomimetic Lipoprotein-Mediated Targeted Nerve Growth Factor Delivery for Traumatic Brain Injury Therapy

**Authors**

*Jialin Huang^1, 2^*†*, Wenye Wang^1, 2^*†*, Yidong Peng^1, 2^*†*, Weiji Weng^1, 2^*†*, Hanyu Wei^1, 2^*†*, Qiyuan Feng^1, 2^, Antian Wang^3^, Minjie Hu^4^, Zhuoran Li^2^, Shenyu Sun^2^, Zhenghui He^1, 2^, Daiwen zhang^1,2^, Wenlan Qi^1,2^, Yuhan Han^1, 2^, Zixuan Ma^1, 2^, Jiyuan Hui^1^, Ru Gong^1^, Yingwei Gao^1, 2^, Yong Lin^1, 2^, Jiyao Jiang^1, 2^, Xiaoling Gao^3^, Junfeng Feng^1, 2^**

**Affiliations**

^1^Department of Neurosurgery, Ren Ji Hospital, Shanghai Jiao Tong University School of Medicine, Shanghai 200127, China.

^2^Shanghai Institute of Head Trauma, Shanghai 200127, China.

^3^Department of Pharmacology and Chemical Biology, Shanghai Universities Collaborative Innovation Center for Translational Medicine, Shanghai Jiao Tong University School of Medicine, Shanghai 200025, China

^4^Department of Radiation Oncology, The First Hospital of Lanzhou University, Lanzhou University, Lanzhou 730000, China.

†: These five authors contributed equally to this work and should be considered co-first authors.

*Corresponding Author: Junfeng Feng, [fengjfmail@163.com](mailto:fengjfmail@163.com)

**Supplementary Table 1.** Characterization of Nc-rHDL@P and its control formulations

| Formulations | Size (nm) | PDI | Ζeta (mV) |
| --- | --- | --- | --- |
| Nc | 274.38±2.31 | 0.14±0.03 | 3.40±0.46 |
| Lipo | 28.75±5.40 | 0.34±0.05 | -51.17±5.56 |
| Nc-Lipo | 52.63±14.35 | 0.40±0.09 | -40.78±0.80 |
| Nc-rHDL | 48.30±5.37 | 0.38±0.01 | -40.83±0.49 |
| Nc-rHDL@P | 45.47±6.57 | 0.26±0.03 | -42.46±0.45 |

Note: Data represent mean ± SD (n = 3).


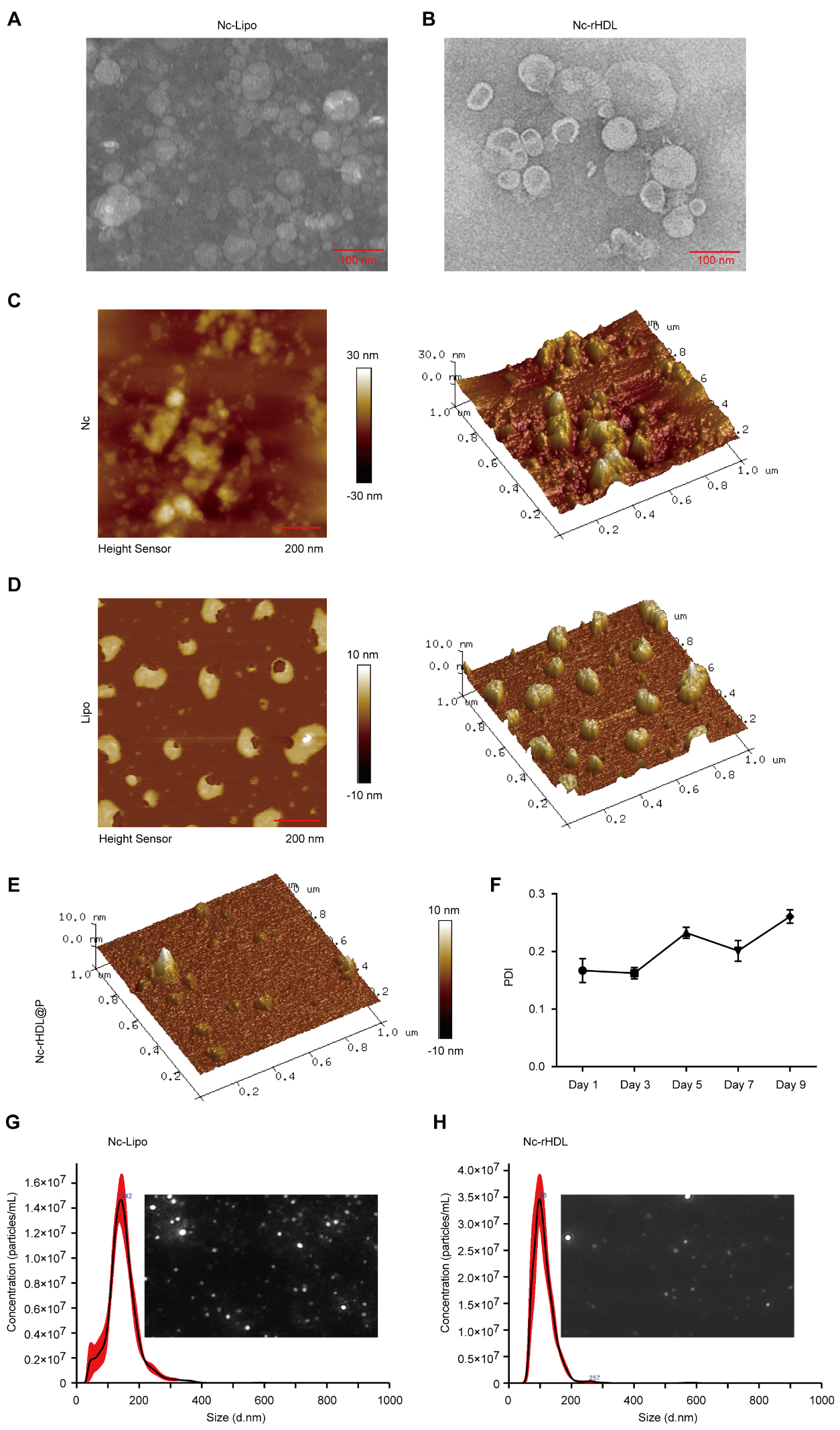
**Figure S1. The characterization of Nc-rHDL@P and its related formulations.** The morphology of Nc-Lipo A) and Nc-rHDL B) under TEM. Scale bar, 100 nm. Topographic images and three-dimensional projections of Nc C), Lipo D) and Nc-rHDL@P E) analyzed by AFM. Scale bar, 200 nm. F) Polydispersity index (PDI) of Nc-rHDL@P measured by DLS. Data represent mean ± SD, n = 3. Particle size distribution and concentration of Nc-Lipo G) and Nc-rHDL H) measured by NanoSight NS300.


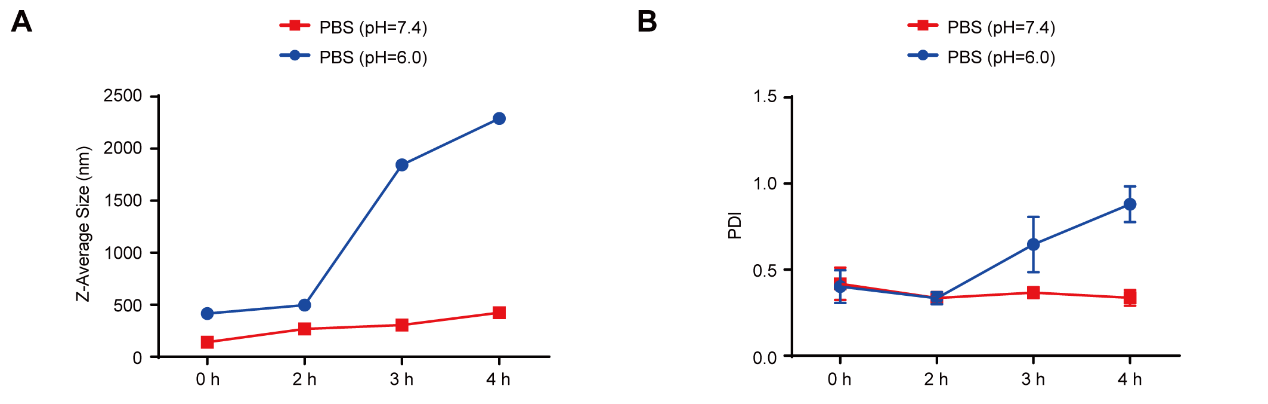


**Figure S2. The characterization of free NGF.** Change in Z–average diameter (A) and PDI (B) of free NGF incubated in PBS buffer with different pH at 37^o^C. Data represent mean ± SD, n = 3.


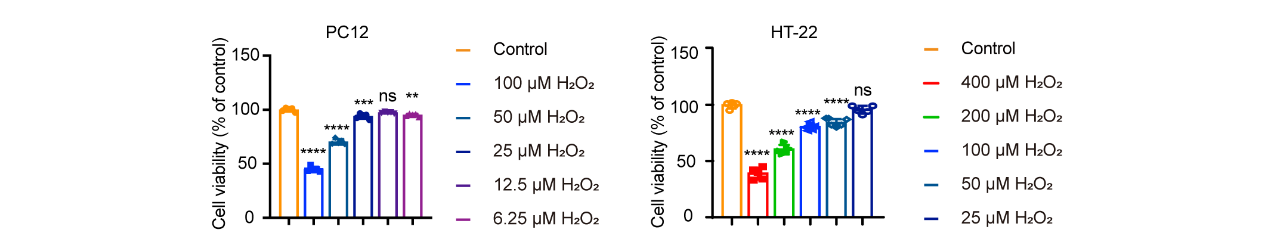


**Figure S3. Oxidative stress model was established in vitro.** PC12 and HT-22 cells were incubated with various concentrations of H_2_O_2_ to investigate cytotoxicity. The control group was defined as 100%. Data represent mean ± SD, n≥3. **p* < 0.05, ***p* < 0.01, ****p* < 0.001, and *****p* < 0.0001. ns, not significant. One-way ANOVA with Bonferroni’s multiple comparisons test was used.


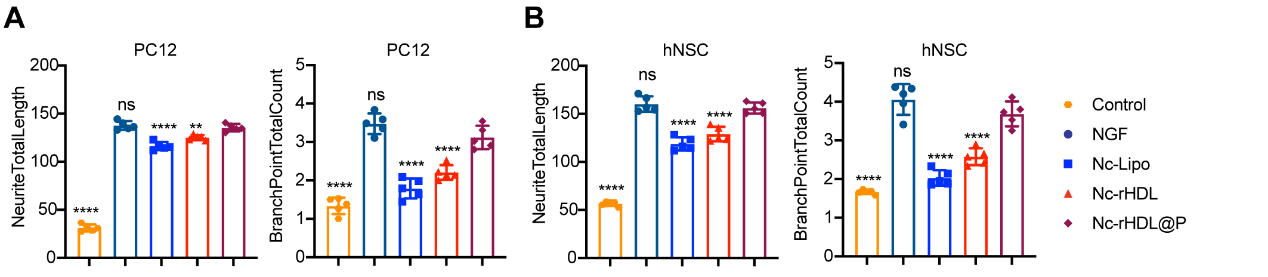


**Figure S4. Nc-rHDL@P promoted the differentiation of PC12 cells and hNSCs.** A) Neurite total length and branch point total count of PC12 cells following 7-day treatment with free NGF and NGF formulations at the NGF dose of 100 ng mL^−1^ by HCS. B) Neurite total length and branch point total count of hNSC cells following 7-day treatment with free NGF and NGF formulations at the NGF dose of 100 ng mL^−1^ by HCS. Data represent mean ± SD, n = 5. **p* < 0.05, ***p* < 0.01, ****p* < 0.001, and *****p* < 0.0001. ns, not significant. One-way ANOVA with Bonferroni’s multiple comparisons test was used.


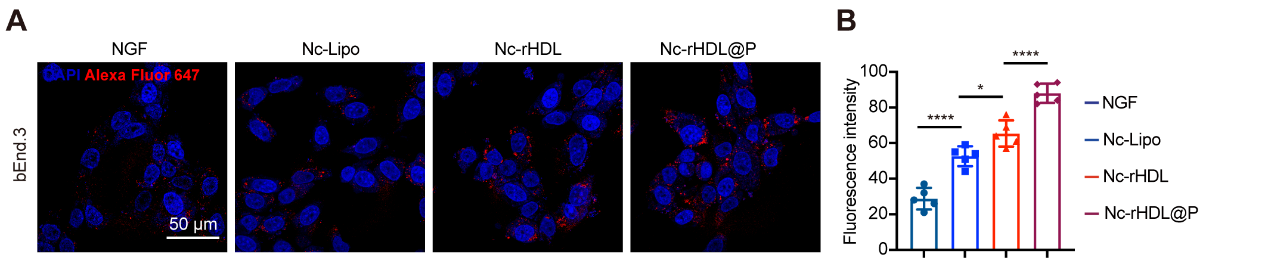


**Figure S5. Nc-rHDL@P efficiently delivered NGF into bEnd.3 cells.** A) Cellular uptake of free NGF and NGF formulations by bEnd.3 cells after 4 h incubation. The concentration of Alexa Fluor 647-labeled NGF was 20 μg mL^−1^. Scale bar, 50 μm. B) Semi-quantitative analysis of the intracellular uptake of Alexa Fluor 647-labeled NGF in bEnd.3 cells. Data represent mean ± SD, n = 5. **p* < 0.05, ***p* < 0.01, ****p* < 0.001, and *****p* < 0.0001. ns, not significant. One-way ANOVA with Bonferroni’s multiple comparisons test was used.


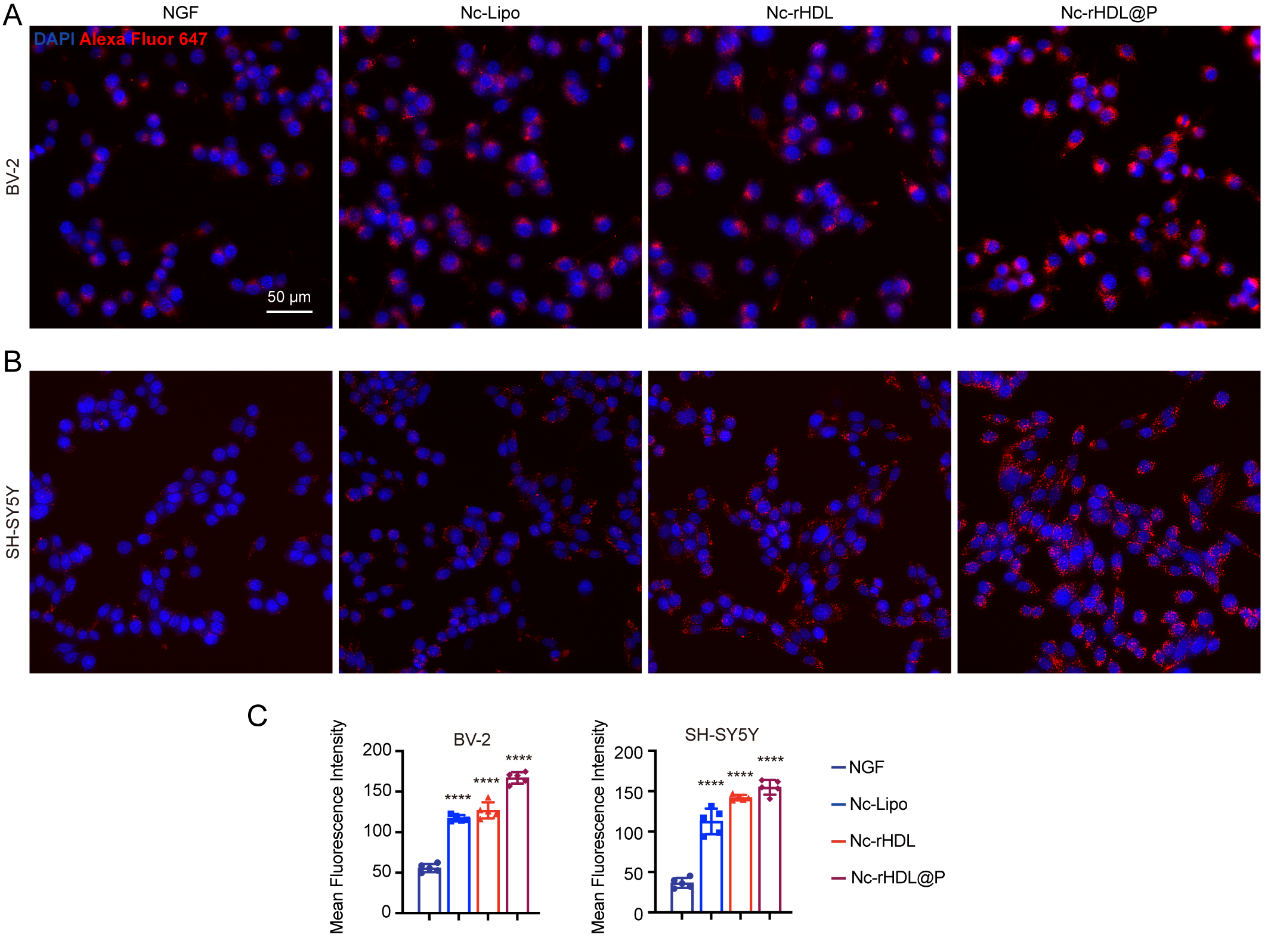


**Figure S6. Nc-rHDL@P efficiently traversed the in vitro BBB model and was taken up by nerve cells seeded in the basal compartment.** A) The cellular uptake of different preparations by BV-2 and SH-SY5Y cells seeded in the basal compartment was examined after 4 h of addition to the apical compartment. Scale bar, 50 μm. B) Semi-quantitative analysis of the intracellular uptake of Alexa Fluor 647-labeled NGF by BV-2 and SH-SY5Y cells. Data represent mean ± SD, n = 5. **p* < 0.05, ***p* < 0.01, ****p* < 0.001, and *****p* < 0.0001. ns, not significant. One-way ANOVA with Bonferroni’s multiple comparisons test was used.


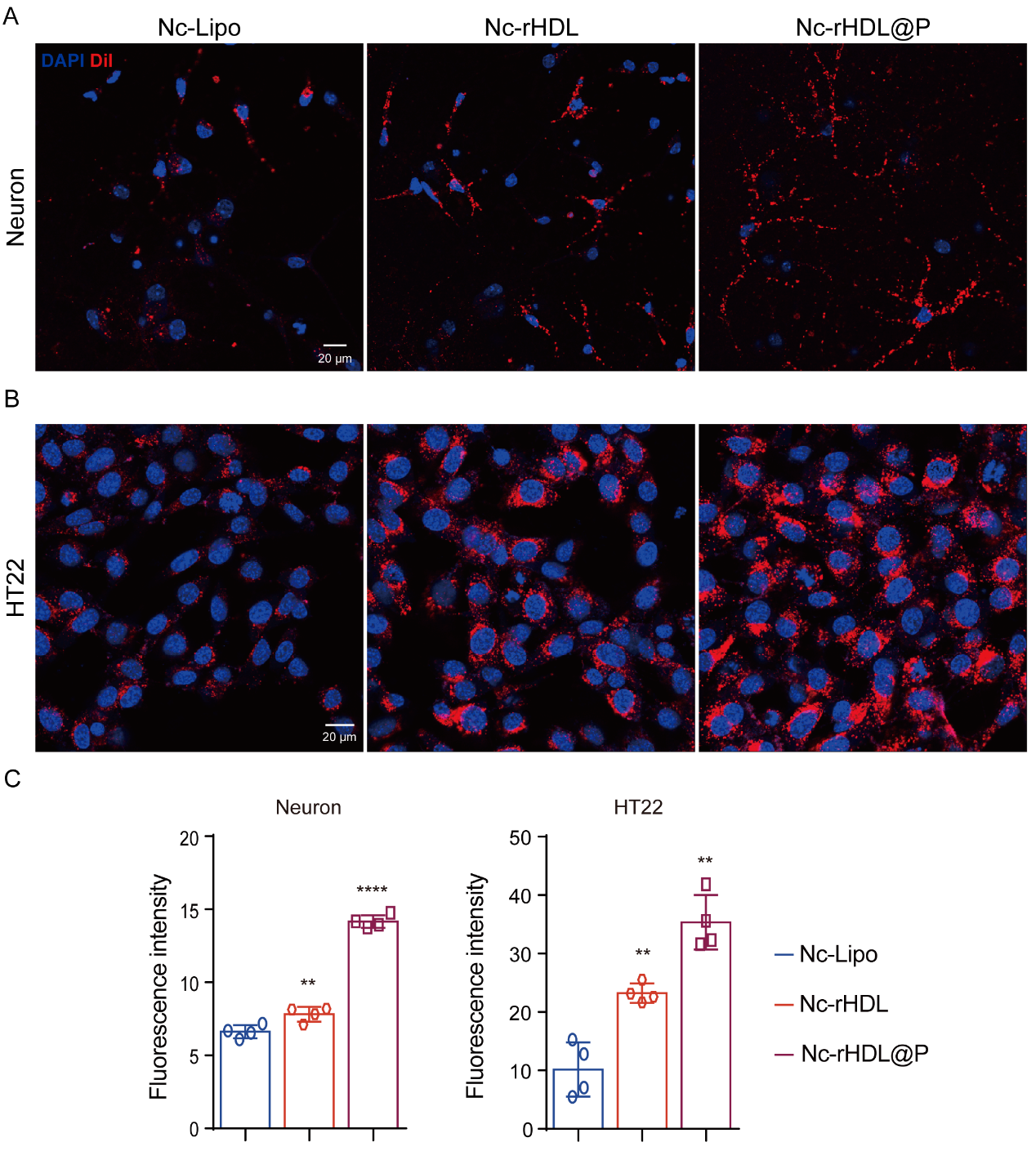


**Figure S7. Nc-rHDL@P exhibited high neurophilicity towards nerve cells.** Cellular uptake of Nc-Lipo, Nc-rHDL, and Nc-rHDL@P by primary neurons A) and HT22 cells B) after 4 h incubation at the DMPC concentration of 10 μg mL^−1^. Red: DiI-labeled lipid membrane. Scale bar, 20 μm. C) Semi-quantitative analysis of the intracellular uptake of DiI-labeled Nc-Lipo, Nc-rHDL, and Nc-rHDL@P. Data represent mean ± SD, n = 4. **p* < 0.05, ***p* < 0.01, ****p* < 0.001, and *****p* < 0.0001. ns, not significant. One-way ANOVA with Bonferroni’s multiple comparisons test was used.


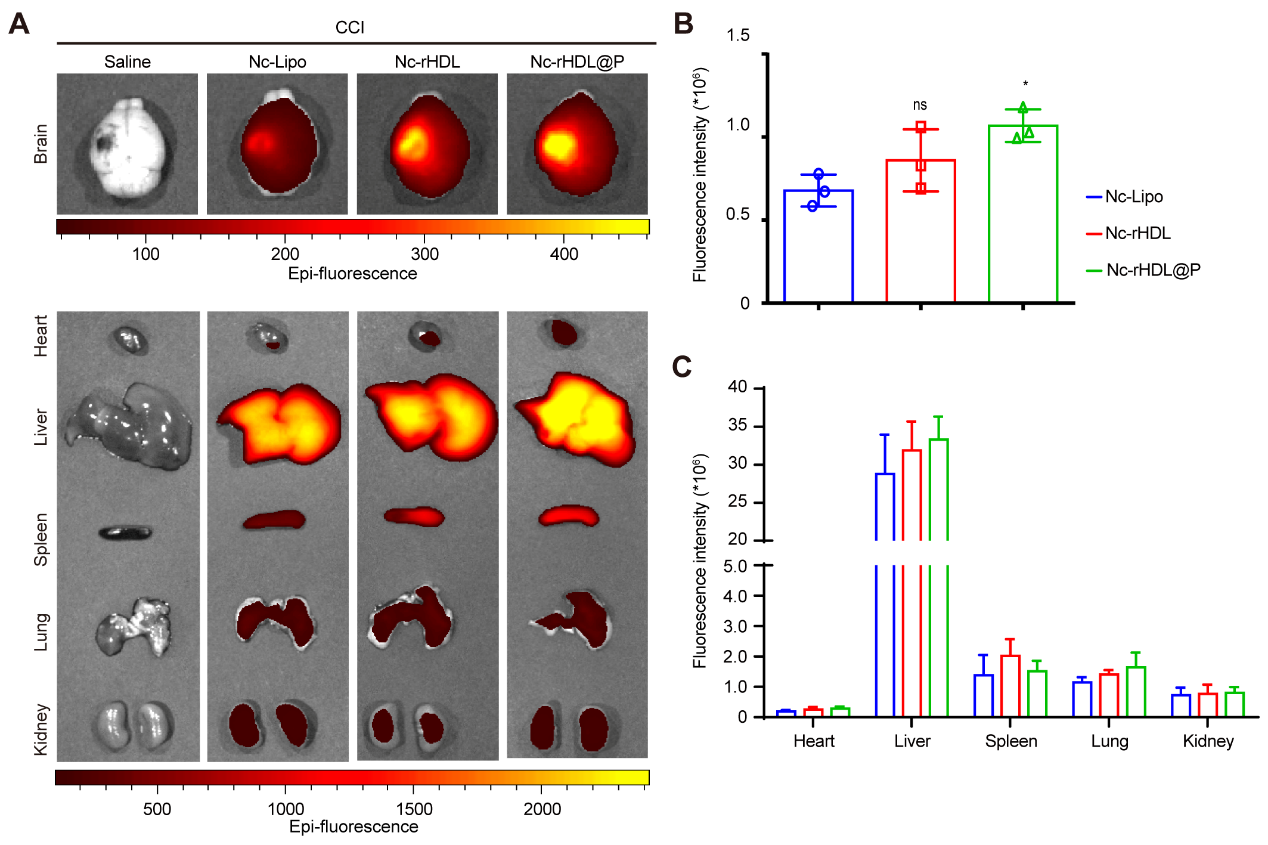


**Figure S8. Nc-rHDL@P efficiently targeted lesion sites in CCI mice.** A) Biodistribution of DiR-labeled NGF formulations at 4 h after administration at the DMPC dose of 5 mg kg^−1^ in CCI model mice. B) Semi-quantitative analysis of the brain distribution of DiR-labeled NGF formulations. C) Semi-quantitative analysis of the organ-distribution of DiR-labeled NGF formulations. Data represent mean ± SD, n = 3. **p* < 0.05, ***p* < 0.01, ****p* < 0.001, and *****p* < 0.0001. ns, not significant. One-way ANOVA with Bonferroni’s multiple comparisons test was used.


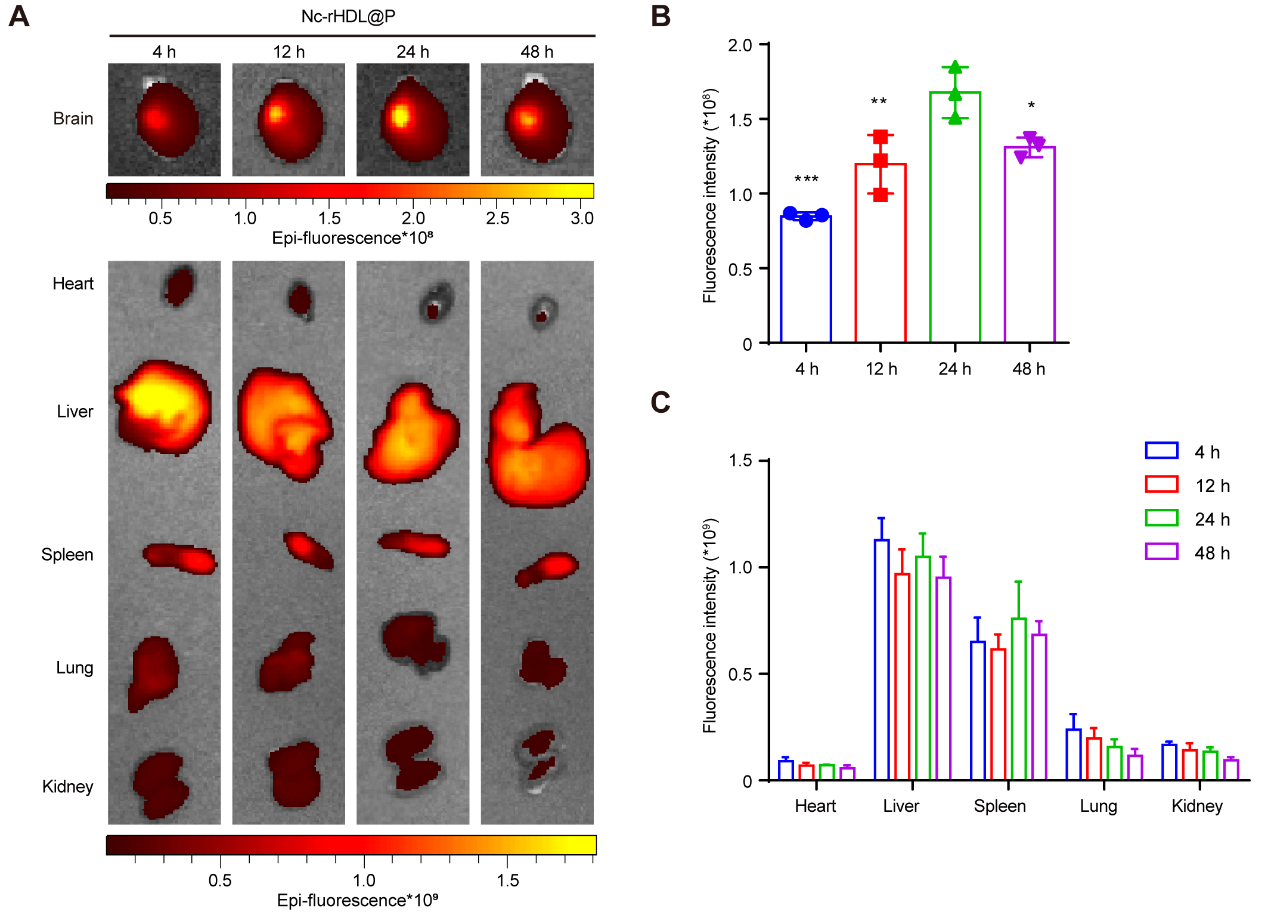


**Figure S9. Biodistribution kinetics of Nc-rHDL@P in CCI model mice.** A) Biodistribution of DiR-labeled Nc-rHDL@P at 4, 12, 24, and 48 h after intravenous administration at the DMPC dose of 5 mg/kg. B) Semi-quantitative analysis of the brain distribution at 4, 12, 24, and 48 h after intravenous injection. (C) Semi-quantitative analysis of the organ accumulation at 4, 12, 24, and 48 h after intravenous injection. Data represent mean ± SD (n = 3). **p* < 0.05, ***p* < 0.01, ****p* < 0.001, and *****p* < 0.0001. ns, not significant. One-way ANOVA with Bonferroni's multiple comparisons test for group comparisons.


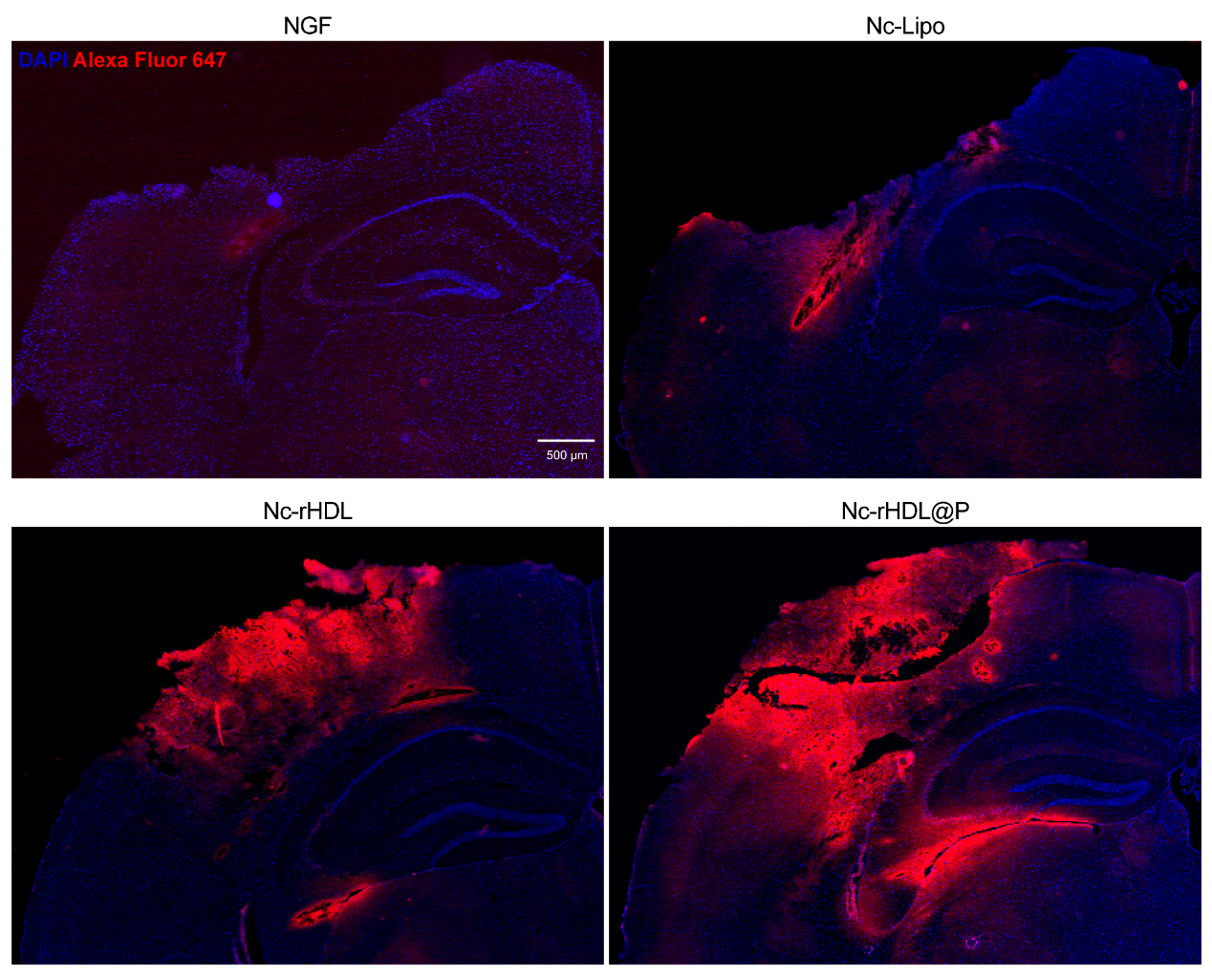


**Figure S10. Nc-rHDL@P efficiently delivered NGF into lesion sites in CCI model mice.** Brain distribution of Alexa Fluor 647-labeled NGF, Nc-Lipo, Nc-rHDL, and Nc-rHDL@P at 4 h after administration at the NGF dose of 2.5 mg kg^−1^ in C57BL/6 mice with CCI. Red: Alexa Fluor 647-labeled NGF. Scale bar, 500 μm.


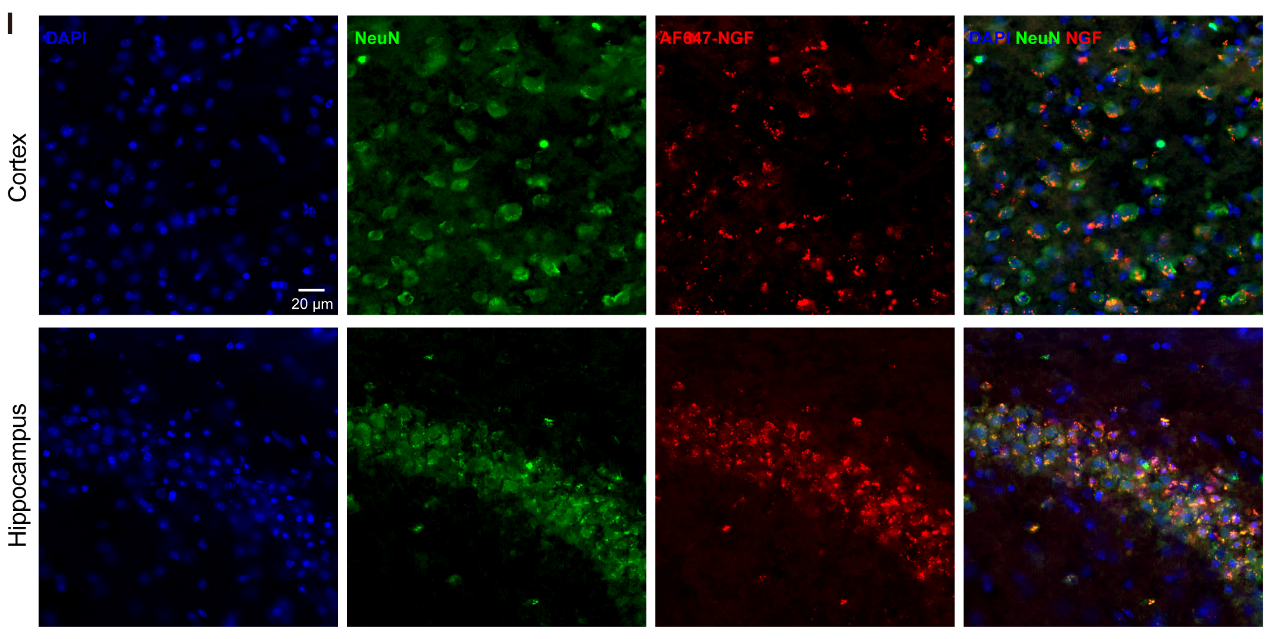


**Figure S11. Nc-rHDL@P achieved neuron-targeted delivery of NGF in CCI model mice.** Brain distribution of Alexa Fluor 647-labeled Nc-rHDL@P at 4 h after administration at the NGF dose of 2.5 mg kg^−1^ in CCI model mice. Red: Alexa Fluor 647-labeled NGF. Green: NeuN^+^ neurons. Scale bar, 20 μm.


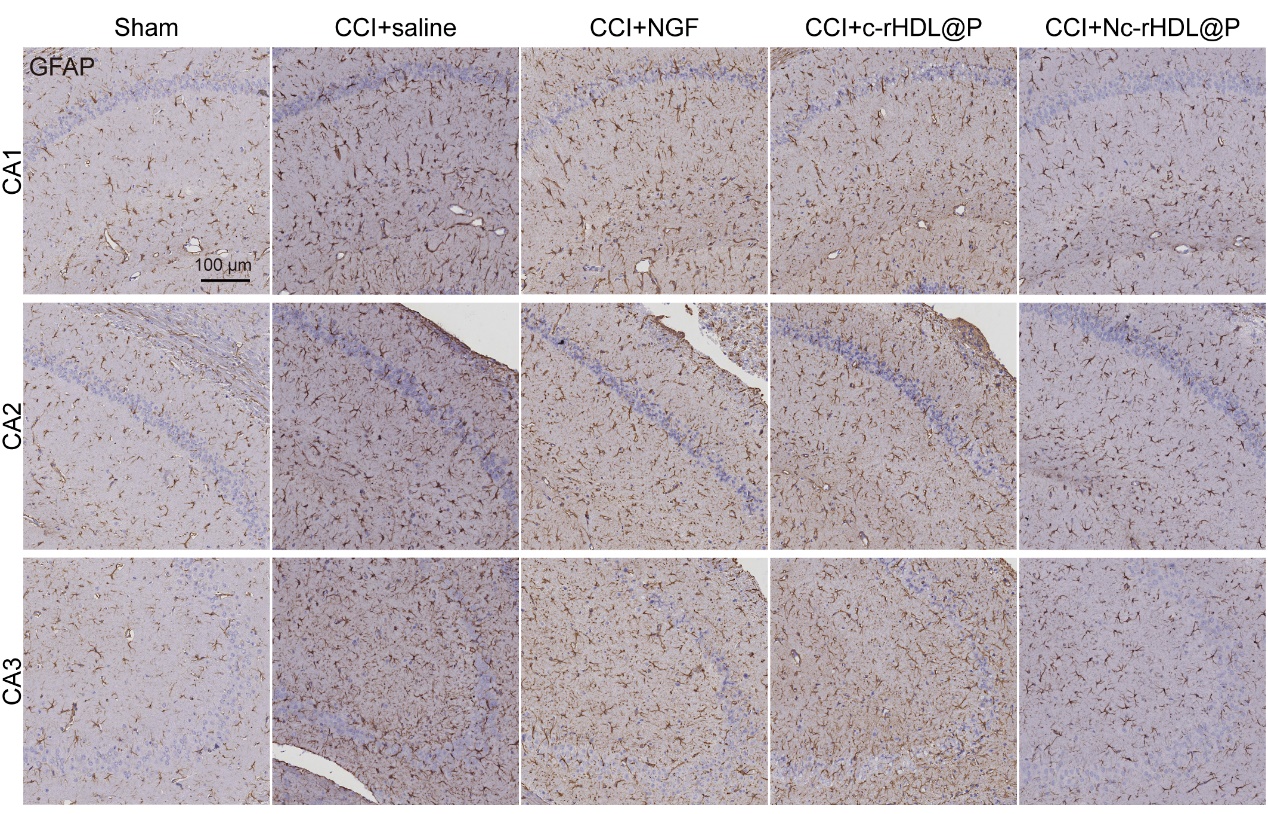


**Figure S12. Nc-rHDL@P improved the inflammatory microenvironment in CCI model mice.** GFAP immunostaining of CA1, CA2, and CA3 in CCI model mice after the treatment of saline, free NGF c-rHDL@P and Nc-rHDL@P. The sham group was not treated with cortical injury. n = 4, Scale bar: 100 μm.


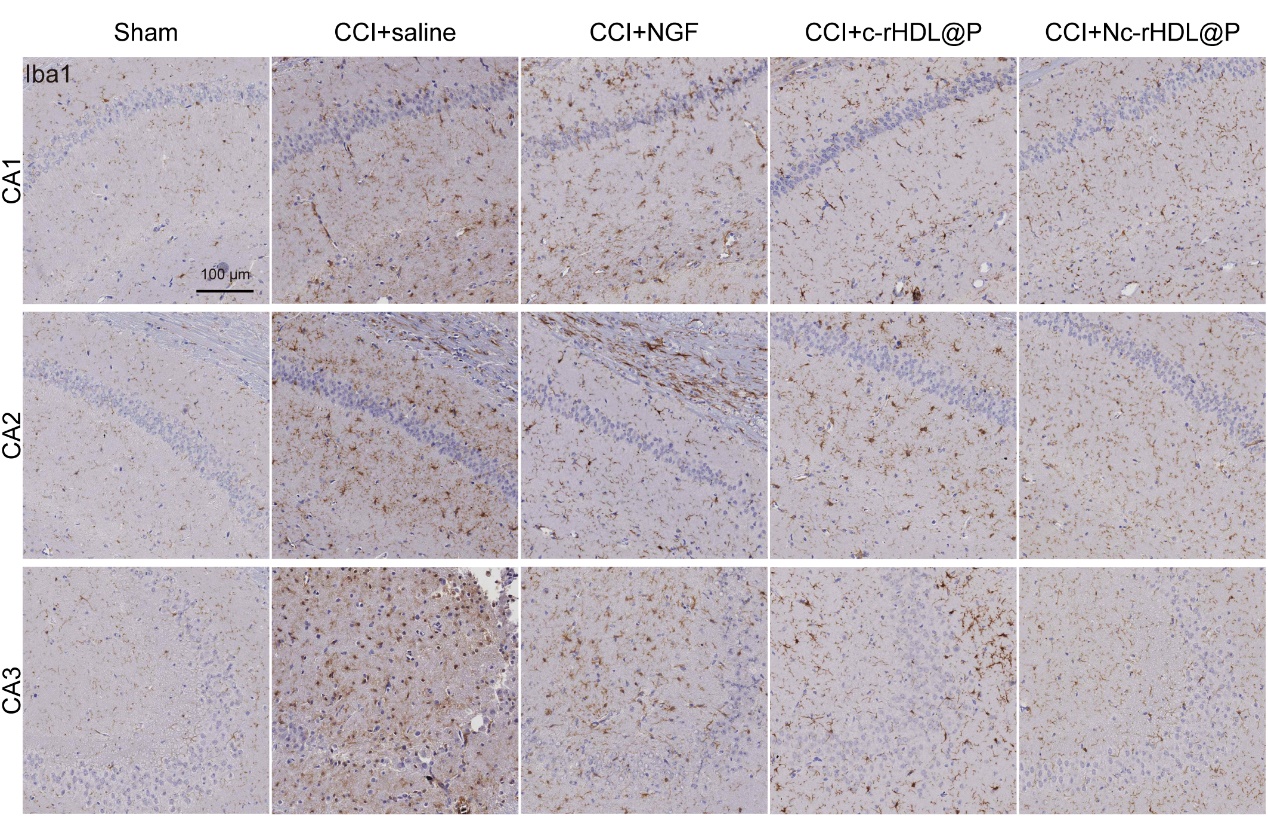


**Figure S13. Nc-rHDL@P improved the inflammatory microenvironment in CCI model mice.** Iba1 immunostaining of CA1, CA2, and CA3 in CCI model mice after the treatment of saline, free NGF, c-rHDL@P, and Nc-rHDL@P. The sham group was not treated with cortical injury. n = 4, Scale bar: 100 μm.


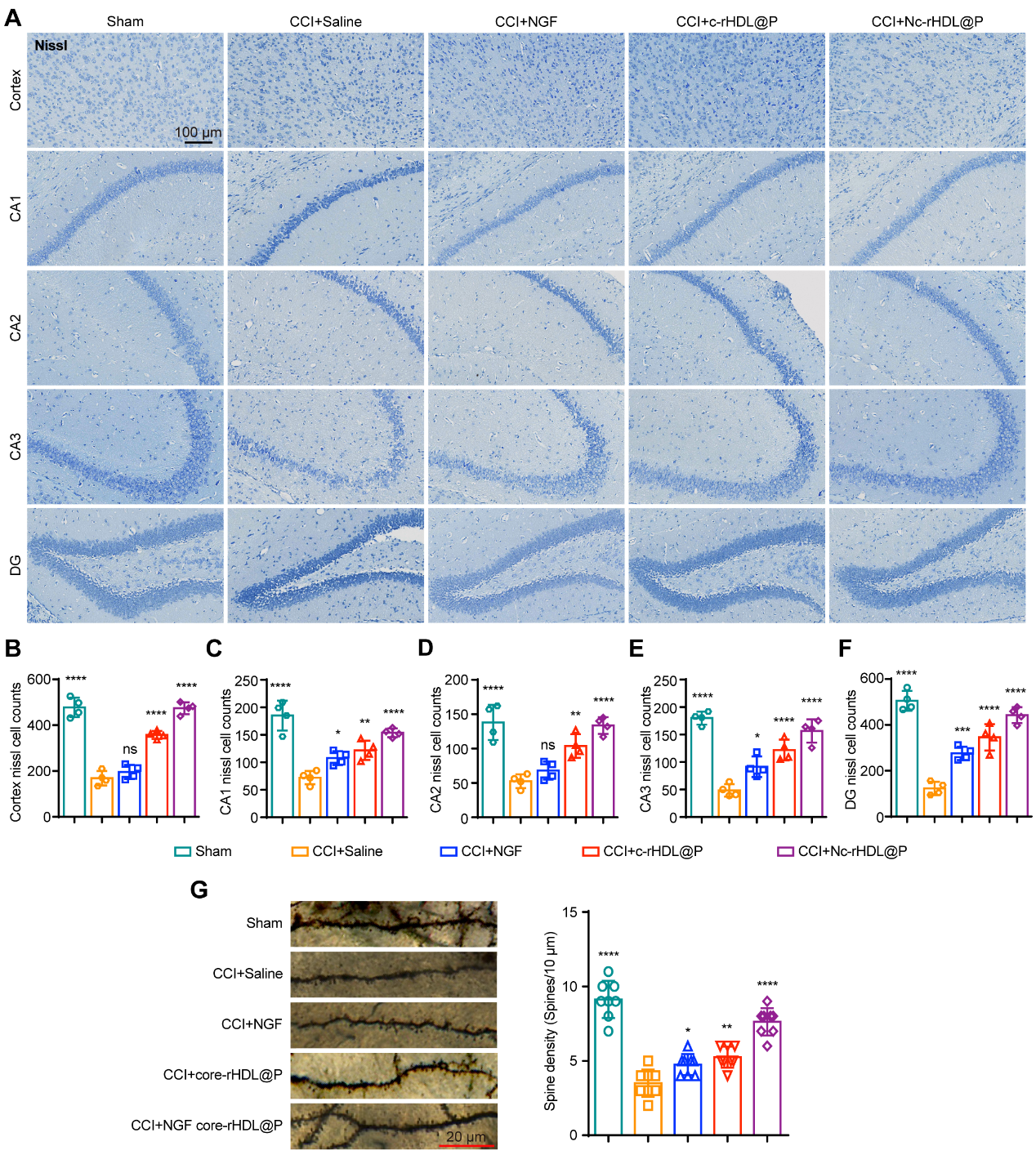


**Figure S14. Nc-rHDL@P attenuated neuropathological damage in CCI model mice.** A) The representative images of Nissl staining of the cortex and subareas of the hippocampus in CCI model mice after treatment with saline, free NGF, c-rHDL@P, or Nc-rHDL@P. Scale bar, 100 μm. B-F) Semi-quantitative analysis of Nissl cell counts. G) The representative images of Golgi-Cox staining dendritic spines of cortical neurons in CCI model mice. ns, not significant. Data represent mean ± SD, n ≥ 3. **p* < 0.05, ***p* < 0.01, ****p* < 0.001, and *****p* < 0.0001. ns, not significant. One-way ANOVA with Bonferroni’s multiple comparisons test was used.


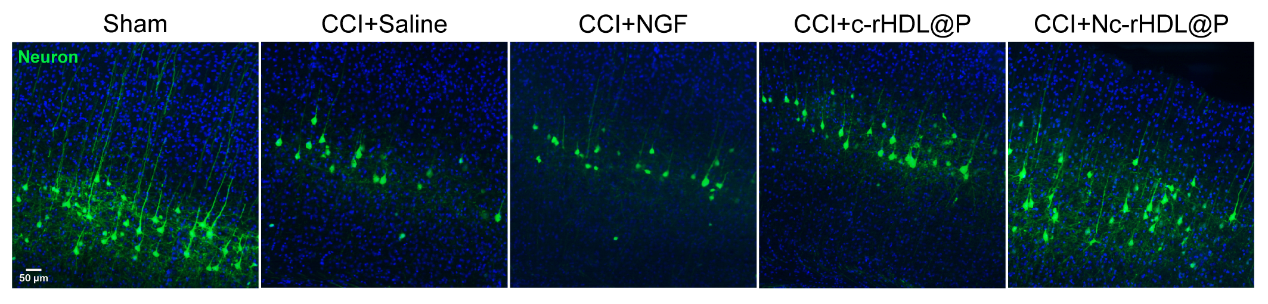


**Figure S15. Nc-rHDL@P promoted neuronal survival and structural maintenance in CCI model mice.** The representative images of cortical neuronal survival and structure in CCI model mice after treatment with saline, free NGF, c-rHDL@P, or Nc-rHDL@P. Scale bar, 50 μm.


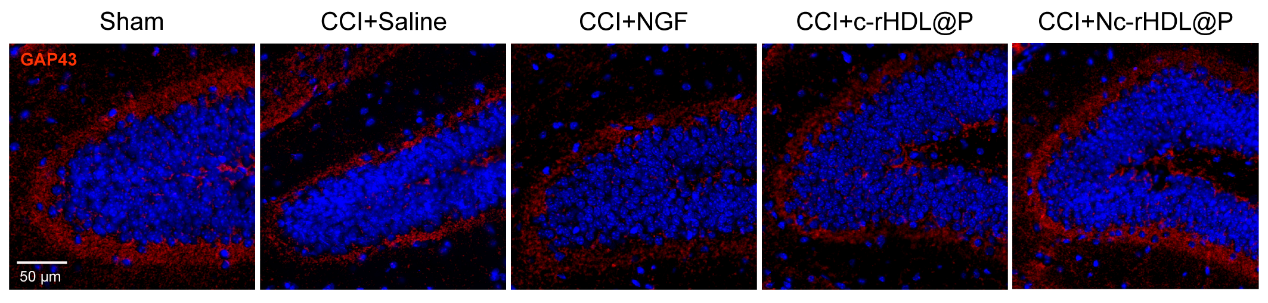


**Figure S16. Nc-rHDL@P enhanced hippocampal GAP43 expression in CCI model mice.** The representative images of hippocampal GAP43 expression in CCI model mice after treatment with saline, free NGF, c-rHDL@P, or Nc-rHDL@P. Scale bar, 50 μm.


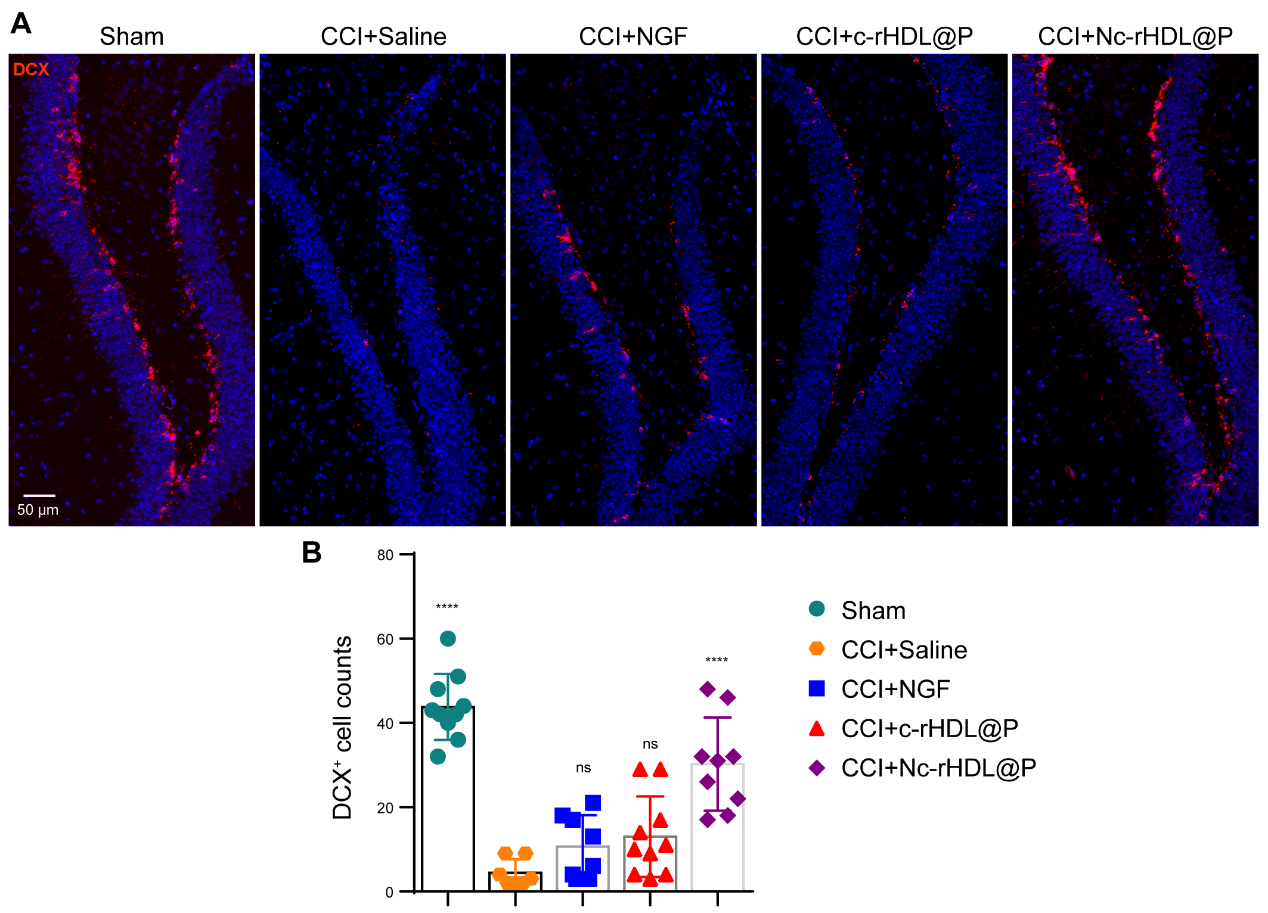


**Figure S17. Nc-rHDL@P enhanced hippocampal DCX expression in CCI model mice.** (A) The representative images of hippocampal DCX expression in CCI model mice after treatment with saline, free NGF, c-rHDL@P, or Nc-rHDL@P. Scale bar, 50 μm. (B) Semi-quantitative analysis of DCX+ cells in the DG after different treatments. Data represent mean ± SD, n ≥ 3. **p* < 0.05, ***p* < 0.01, ****p* < 0.001, and *****p* < 0.0001. ns, not significant. One-way ANOVA with Bonferroni’s multiple comparisons test was used.


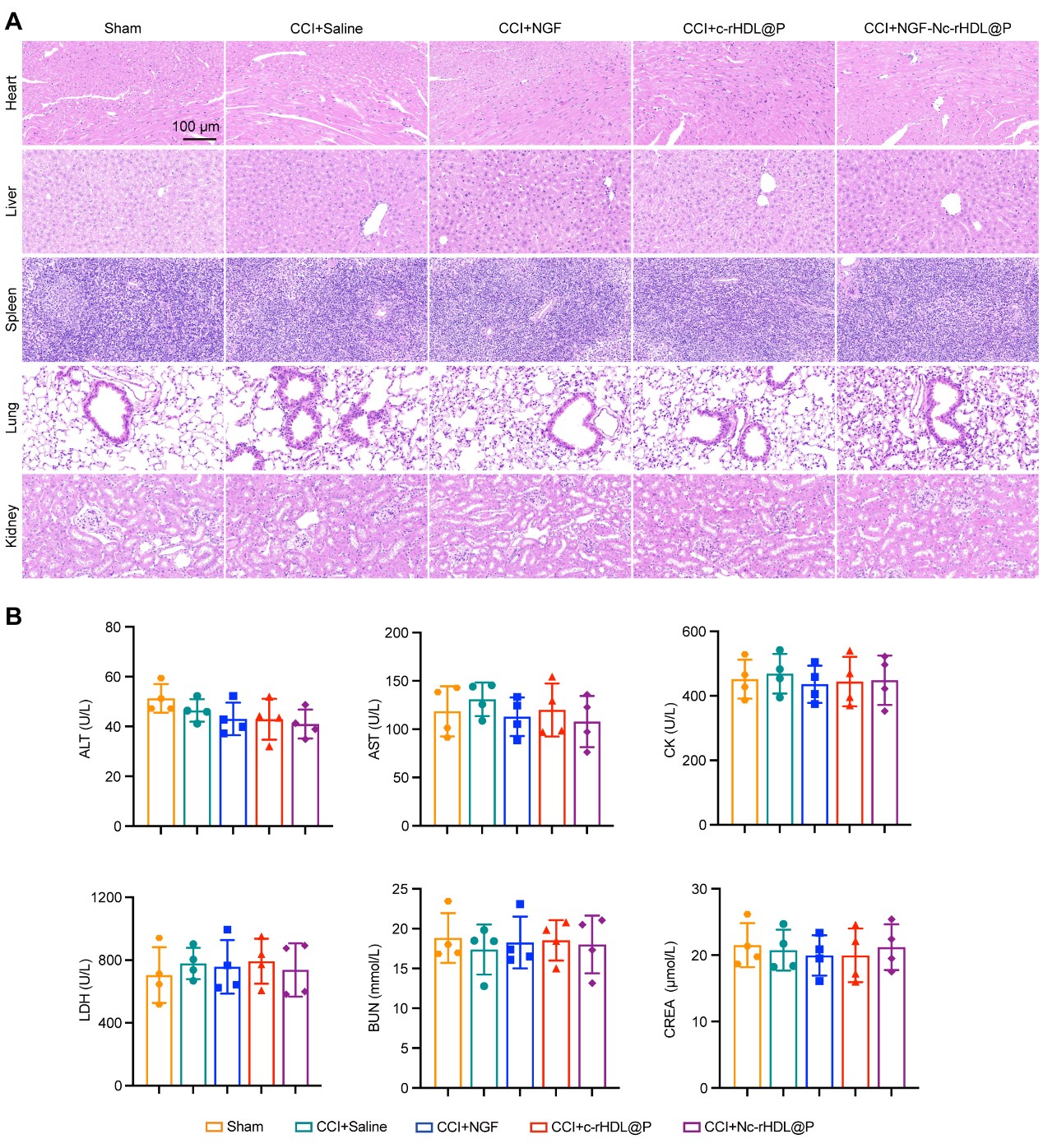


**Figure S18. Evaluation of the safety of Nc-rHDL@P in CCI model mice.** A) Organ morphology of CCI model mice after treatment with free NGF or NGF formulations. Scale bar, 100 μm. B) Serum biochemistry assay of CCI model mice after treatment. Data represent mean ± SD, n = 4.
